# Immobilized Gold Nanoparticles Prepared from Gold(III)-Containing Ionic Liquids on Silica: Application to the Sustainable Synthesis of Propargylamines

**DOI:** 10.3390/molecules23112975

**Published:** 2018-11-14

**Authors:** Raquel Soengas, Yolanda Navarro, María José Iglesias, Fernando López-Ortiz

**Affiliations:** Área de Química Orgánica, Research Centre CIAIMBITAL, Universidad de Almería, Carretera de Sacramento s/n, 04120 Almería, Spain; yng453@ual.es (Y.N.); mjigle@ual.es (M.J.I.)

**Keywords:** gold, nanoparticles, multicomponent reaction, supported catalyst, propargylamine, HRMAS

## Abstract

A cycloaurated phosphinothioic amide gold(III) complex was supported on amorphous silica with the aid of an imidazolium ionic liquid (IL) physisorbed in the SiO_2_ pores (SiO_2_–IL) and covalently bonded to the SiO_2_ (SiO_2_@IL). Gold(0) nanoparticles (AuNPs) were formed in situ and subsequently immobilized on the SiO_2_–IL/SiO_2_@IL phase. The resulting catalytic systems Au–SiO_2_–IL and Au–SiO_2_@IL promoted the solvent-free A^3^ coupling reaction of alkynes, aldehydes, and amines in high yields under solvent-free conditions with very low catalyst loading and without the use of additives. The Au–SiO_2_@IL catalyst showed good recyclability and could be reused at least five times with yields of propargylamines of ≥80%. This synthetic method provides a green and low cost way to effectively prepare propargylamines. Additionally, ^31^P high resolution magic angle spinning (HRMAS) NMR spectroscopy is introduced as a simple technique to establish the Au loading of the catalyst.

## 1. Introduction

Propargylamines are versatile synthetic intermediates and important structural elements of numerous natural and synthetic products that show a wide range of biological activities [1,2,3,4,5]. Traditionally, these compounds have been synthesized by the nucleophilic attack of metal acetylides on imines or their derivatives, but these reagents are used in stoichiometric amounts, are highly moisture sensitive, and require strictly controlled reaction conditions [6]. An alternative atom-economical procedure for the synthesis of propargylamines is the one-pot, three-component, metal-catalyzed reaction of an alkyne, an aldehyde, and an amine, the so-called A^3^ coupling [7]. Homogeneous catalysts based on a large number of transition metal ions have been used for the A^3^ coupling reaction, including Cu [8,9,10,11,12], Ag [13,14,15,16], Cu/Ru bimetallic systems [17,18], Zn [19,20,21], Fe [22,23], In [24,25], Ir [26,27], Hg [28], Ni [29], Rh [30], Mn [31], Co [32], Bi [33], and Cd [34]. Over the past few years, increasing attention has been paid to the catalytic performance of Au in promoting the A^3^ coupling reaction, including Au(I) and Au(III) salts and organic Au complexes [35,36,37,38]. Despite cationic Au species showing very high catalytic activity, their rapid reduction under alkyne activation conditions is an important drawback [39,40,41]. It should also be noted that homogeneous catalysts suffer from the difficulty of catalyst separation and reuse as well as the problem of product contamination with the metal. The contamination of products with metals is a major problem in pharmaceutical development, since trace amounts of metal contamination could have unwanted effects on biological systems. In order to overcome these limitations, the use of heterogenizing homogeneous Au catalysts in organic synthesis has attracted a large amount of attention because the advantages of both homogeneous and heterogeneous catalysis can be gathered in the system [42,43,44]. In this regard, a few heterogeneous and recyclable gold catalysts have been reported for A^3^ coupling reactions [45,46]. During the last decade, supported gold(0) nanoparticles (AuNPs) have been explored as sustainable and competitive alternatives to gold complexes for A^3^ coupling reactions [47,48,49]. Despite AuNPs promoting the A^3^ coupling in their pure form, they tend to agglomerate, which limits their efficiency in catalytic processes [48]. On the contrary, supported AuNPs enhance the activity and selectivity of reactions and could be easily heterogenized [48,49]. Several nanocatalysts based on AuNPs and different supports have successfully been employed in A^3^ coupling reactions. Thus, clays [50,51,52], nanocomposites [53,54], graphenes [55], metal-organic frameworks [56,57,58,59], resins [60], polymeric materials [61], magnetic supports [62,63,64], and biomaterials [65,66] have been used in this reaction. However, there is still much room for improvement, as some of the reported procedures require the use of harmful organic solvents, long reaction times, or a laborious and time-consuming support preparation process.

In recent years, ionic liquids [67,68] have attracted much recent interest in transition metal catalysis, since they can act as co-catalysts and enhance the activity of metallic species present in catalytic systems [69]. As a consequence of their pre-organized structure with hydrogen bonds connecting cations and anions, the enhanced catalytic activity and stability of metal complexes and nanoparticles in ionic liquids (ILs) is related to the surface electronic stabilization of imidazolium aggregates forming protective layers. In recent years, the concept of supported ionic liquid phase catalysis (SILPC) has gained much attention. In this regard, it has been proven that a thin film of the ionic liquid supported on a porous material is suitable for the stabilization of catalytically active complex or metal nanoparticles [70,71]. Grafting ILs onto the surfaces of solid materials not only reduces the use of the relatively expensive ILs, but also immobilizes the metal complexes or nanoparticles, facilitating their recovery and recycling [72,73,74]. SILP catalysts have been widely applied in palladium-catalyzed cross-coupling reactions, but only very limited study has been done on SILP gold catalysis. A recent report described the immobilization of gold(III) on poly(ionic liquid) coated on magnetic nanoparticles to produce a highly active, stable, and recoverable catalyst for the synthesis of propargylamines via A^3^ coupling reactions carried out with water as a solvent [75]. Gold(III) nanoparticles supported on periodic mesoporous silica containing ionic liquid proved to be an efficient catalyst for the A^3^ synthesis of propargylamines with chloroform as a solvent at a loading of 0.2 mol% of gold [76]. Interestingly, under solvent free conditions, the reaction yield decreased to 50%. Furthermore, the performance of the analogous reaction using gold(0)-supported nanoparticles showed a conversion of less than 15%. Despite the evident interest in performing the reaction under solvent-free conditions from a sustainability point of view, there are very limited examples in the literature [77,78,79]. AuCl_4_^−^ dispersed over ionic liquids grafted on MCM-41 catalyst was used as a catalyst to promote the solvent-less A^3^ coupling reaction [80]. However, the procedure suffered from a significant decrease in the catalytic activity in successive reaction cycles. This result can be explained on the basis of gold leaching. To sum up, the synthesis of propargylamines using eco-friendly and reusable catalysts under solvent-free conditions is still a thrust area in the chemical field.

We have previously reported the solvent-less synthesis of propargylamines via A^3^ coupling using a gold(III) cycloaurated phosphinothioic complex as the pre-catalyst [81]. The real catalysts were identified as Au(I) nanoparticles. Attempts to isolate the Au(I)NPs produced their reduction to Au(0)NPs and deactivation. We reason that the immobilization of gold nanoparticles in supported ionic liquids would offer unique possibilities for the Au-catalyzed A^3^ coupling reaction. In the work herein presented, we describe the stabilization of Au(0)NPs generated from a cycloaurated phosphinothioic complex on an imidazolium ionic liquid supported on amorphous silica. The in situ formed Au–SiO_2_@IL maintains their catalytic activity in the solvent-free three-components synthesis of propargylamines. Moreover, the AuNPs can be easily recovered from the catalytic mixture and reused with almost unchanged activity for five cycles. In addition, the use of ^31^P HRMAS NMR spectroscopy to study the pre-catalytic system is introduced for the first time.

## 2. Results and Discussion

**Scheme 1:**
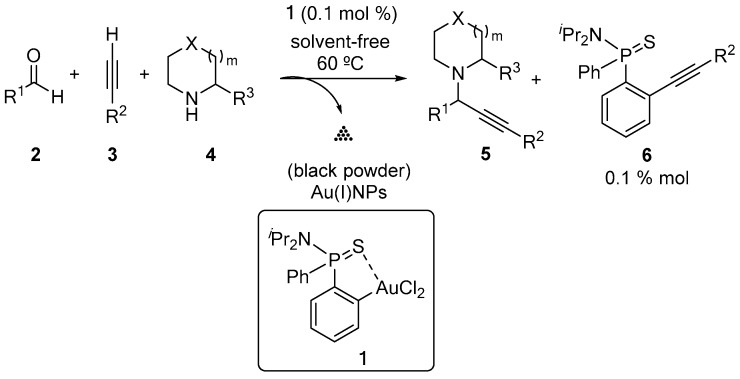
A^3^ coupling reaction catalyzed by Au(III) complex **1**.

Previous results from our group showed that the *ortho*-substituted phosphinothioic amide (dppta) gold(III) complex **1** acts as a pre-catalyst in the highly efficient synthesis of propargylamine **5** via A^3^ coupling reactions under very mild, solvent-free conditions [81]. During the course of the reaction, the Au(III)-complex was transformed into the Sonogashira-type *o*-alkynylphosphinothioic amide **6** with generation of Au(I) nanoparticles, which are the real catalysts (Scheme 1). In order to reuse the catalysts, the NPs were isolated through solvent washing in the air and centrifugation. This procedure led to Au(0)NPs showing a large decrease in activity in the A^3^ coupling synthesis of **5** (yield < 20%). To overcome this limitation, we proposed that the SILPC methodology could be applied to prepare highly-active AuNPs stabilized by the immobilized ionic liquid. The resulting heterogeneous catalysts would facilitate the recyclability. Immobilization of the ionic liquid was studied using two different approaches: (i) physical adsorption of ionic liquids and (ii) covalent bonding via the attachment of ionic liquid fragments to the support.

### 2.1. Physical Confinement of the Ionic Liquid and Complex ***1*** on the Silica Surface

We started our study by investigating a facile physical immobilization of the Au catalyst in an ionic liquid in silica pores for use in sustainable A^3^ coupling reactions. The procedure of immobilization was quite simple [82]; a suspension of spherical amorphous silica in a solution of (dppta)AuCl_2_ complex **1** in [bmim]PF_6_ and CH_3_CN was evaporated to dryness and washed with diethyl ether to afford a powdery and free-flowing immobilized catalyst. The system formed by pre-catalyst **1** and the SiO_2_–IL support [(dppta)AuCl_2_–SILP] was characterized by IR spectroscopy and HRMAS NMR.

**Figure 1:**
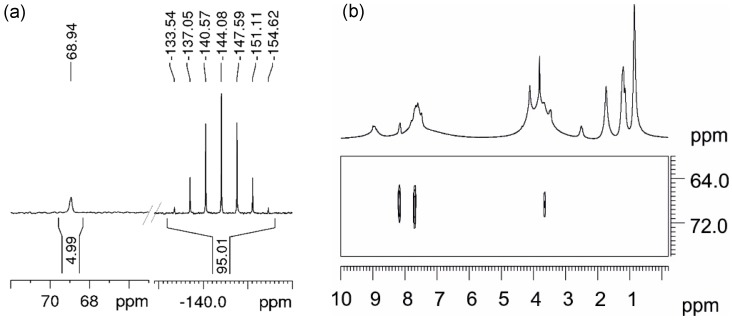
(**a**) ^31^P HRMAS NMR (202.46 MHz) and (**b**) ^1^H, ^31^P HRMAS HMQC (500.13 MHz) spectra of (dppta)AuCl_2_–SiO_2_–[bmim]PF_6_.

Infrared analysis (IR) showed the characteristic bands of the silica support at 3459 cm^−1^ and 1094 cm^−1^, belonging to the Si−OH stretch and the Si−O−Si stretch, respectively. In addition, the bands at 835 cm^−1^ and 1642 cm^−1^, assigned to the stretching vibrations of PF_6_^−^ and C=N respectively, clearly indicated the presence of the ionic liquid [bmim]PF_6_ within the silica support (Figure S1). Blümel and co-workers demonstrated that HRMAS methods can be applied to the acquisition of ^1^H, ^13^C, and ^14^N NMR spectra of ionic liquids immobilized on silica, including two-dimensional ^1^H, ^1^H COSY and ^1^H, ^13^C HMQC correlations [83]. The ^1^H HRMAS NMR spectrum of a DMSO-*d*_6_ suspension of the supported Au(III) complex (1% wt) showed reasonably well-resolved signals for the IL protons and the isopropyl groups of complex **1** if one considers the large amount of silica (1 g) compared with the IL (100 mg) and complex **1** (11 mg) used (Figure S2). Interestingly, the presence of **1** could be also detected in the ^13^C HRMAS NMR spectrum despite the small amount present (Figure S3). The analysis of these spectra in combination with the ^1^H, ^1^H COSY (Figure S4) and ^1^H, ^13^C HSQC-edited (Figure S5) correlations provided the assignment of the ^1^H and ^13^C signals of IL and **1** (see experimental). The phosphorus-containing complex **1** and ionic liquid [bmim]PF_6_ allowed the use of ^31^P-NMR spectroscopy as a simple tool to determine the loading of gold into the solid support. Figure 1a shows the ^31^P HRMAS NMR spectrum of (dppta)AuCl_2_–SiO_2_–[bmim]PF_6_ measured in DMSO-*d*_6_. The integrals of the singlet arising from **1** and the septuplet originated by the PF_6_^-^ moiety (^1^*J*_PF_ = 706.2 Hz) provided a **1**:IL ratio of 1:19, which was in excellent agreement with the experimental mass balance used. Interestingly, and in sharp contrast to the ^1^H HRMAS NMR signals (Figure S2), relatively narrow signals were obtained for both pre-catalyst **1** and the counteranion of the IL. These features can be attributed to differences in mobility between species. Broad signals in the ^1^H HRMAS NMR spectrum arose from restricted mobility due to the grafting of the IL to the silica (short transverse relaxation times, *T*_2_), whereas the higher mobility of **1** and PF_6_^−^ made it feasible to obtain high resolution ^31^P HRMAS NMR signals [84]. Furthermore, it was possible to measure a ^1^H, ^31^P HRMAS HMQC spectrum optimized for the detection of long-range scalar couplings. The expected correlations of the ^31^P signal of **1** with aromatic protons and the methine protons of the isopropyl groups were easily detected (Figure 1b). To the best of our knowledge, these are the first examples of the application of ^31^P HRMAS NMR techniques to characterize dispersed substrates in ILs. Solid-state ^31^P MAS NMR studies of a number of SILP catalysts have been reported [85,86].

First, the synthesis of propargylamines under the supported catalyst was assessed in various conditions, and the results are compiled in Table 1. Thus, 1 equiv of benzaldehyde **2a** was allowed to react with 1 equiv of piperidine **4a** and 1.5 equiv of trimethylsilylacetylene **3a** in the presence of variable amounts of the supported catalyst under solvent-free conditions at 60 °C for 6 h under a nitrogen atmosphere to afford propargylamine **5a**. The reactions proceeded with excellent conversions (Table 1, entries 1–2), similar to those reported for the unsupported phosphinothioic amide gold(III) complex; these are included in Table 1 for comparison (entries 4 and 5). Using 1 mol% of supported catalyst afforded a quantitative yield of **5a** (Table 1, entry 1). Decreasing the catalyst loading to 0.5 mol% gave an excellent yield of 98% of **5a** (Table 1, entry 2). The catalyst loading could be further decreased to 0.1 mol% as long as the reaction time was increased to 8 h. Under such conditions, propargylamine **5a** was formed with a 97% yield (Table 1, entry 3).

The work-up procedure was very simple: the supernatant liquid was separated and the remaining solid was thoroughly washed with *n*-pentane. As expected, the ^31^P-NMR spectra of the crude reaction mixtures showed only one signal at δ 62.8 ppm, corresponding to the Sonogashira product **6a** (R^2^ = tetramethylsilane (TMS) in Scheme 1), indicating that complex **1** was fully transformed in situ to the catalytically active Au nanoparticles, which were efficiently immobilized by the supported ionic liquid phase.

In order to prove this hypothesis, we achieved the characterization of the solid using the scanning electron microscopy (SEM), transmission electron microscopy (TEM), and X-ray photoelectron spectroscopy (XPS) methods.

**Figure 2:**
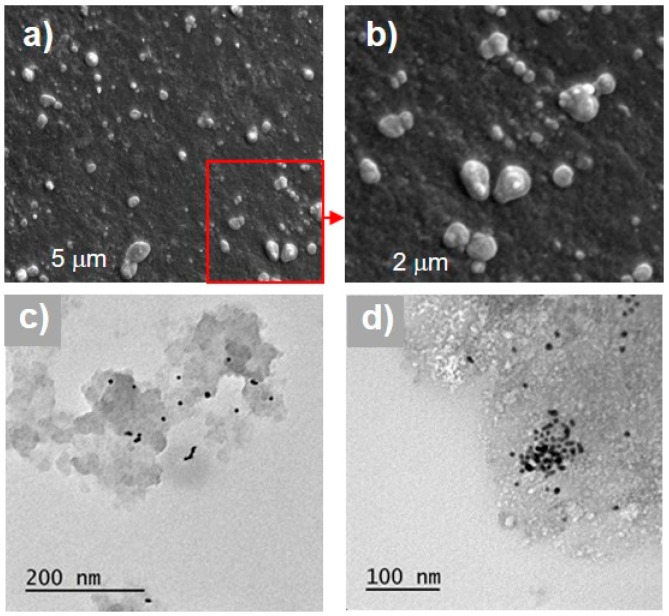
Scanning electron microscopy (SEM) (**a**,**b**) and transmission electron microscopy (TEM) (**c**,**d**) images of AuNPs formed in the A^3^ synthesis of **5a** in the presence of 0.1 mol% of (dppta)AuCl_2_–SiO_2_–[bmim]PF_6_ under solvent-free conditions.

Adsorption of the IL (bright spots) on the pores of the silica was readily observed in the SEM images of Au–SiO_2_–IL system (Figure 2a,b). The TEM image showed the AuNPs immobilized on an amorphous matrix with a narrow size distribution with a diameter of about 7.6 nm (Figure 2c,d). A similar pattern was observed in the Au(I)NPs formed in the absence of the SiO_2_–IL support [81].

**Figure 3:**
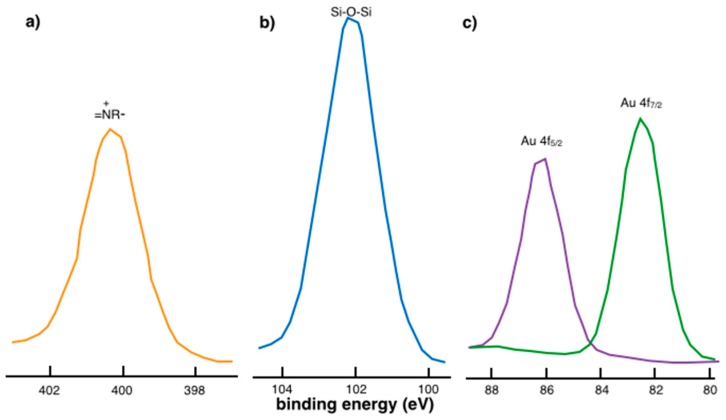
Core level regions X-ray photoelectron spectroscopy (XPS) spectra of the supported AuNPs for (**a**) N 1s, (**b**) Si 2p, and (**c**) Au 4f.

In the full XPS spectrum of AuNPs generated in the synthesis of **5a** after one catalytic run, the peaks corresponding to Au 4f, C 1s, Cl 2s, N 1s, O 1s, and Si 2p and 2s were clearly observed (Figure S6). The XPS spectrum of the N 1s core level region for the supported AuNPs showed a peak at 401.9 eV, which corresponds to the bonding energy of the quaternary nitrogen of the ionic liquid (Figure 3a and Figure S7) [87]. In addition, the high-resolution Si 2p XPS spectrum displayed a peak at 102.2 eV that was attributed to the Si–O–C bonds (Figure 3b and Figure S8) [88]. Finally, the XPS spectrum in the Au 4f region showed two intense doublets at 82.7 and 86.4 eV for Au 4f_7/2_ and Au 4f_5/2_, respectively (Figure 3c and Figure S9), comprising the only Au species in the material and confirming that, during the catalysis, the Au(III) complex was fully reduced, leading to the real catalysts, i.e., the Au(0)NPs, being dispersed on the supported ionic liquid phase of [bmim]PF_6_ encapsulated in silica (Au–SILP). As mentioned previously, Au(I)NPs were identified as the active catalysts in the unsupported A^3^ synthesis of **5a** [81] (see Scheme 1), and the manipulation of the NPs in the air led to deactivated Au(0)NPs. The results above indicate that the deactivation most probably arose from agglomeration [48], a process inhibited when the Au(0)NPs were generated in the supported IL.

With the optimized reaction conditions in hand, we extended our studies to different combinations of aldehydes, amines, and alkynes (Table 2). The electron-withdrawing nature of the chlorine of *p*-chlorobenzaldehyde did not have any effect on the reaction rate, so 94% conversion to **5b** was achieved in 8 h (entry 2). However, the electron-donating group of *p*-methoxybenzaldehyde slowed the reaction significantly, as evidenced by the formation of **5c** with 83% conversion in 14 h (entry 3). Phenylacetylene reacted slightly more slowly to give **5d** with 94% conversion in 8 h (entry 4). Electron-withdrawing groups in the alkyne also produced a decrease in the reaction rate. The use of 4-fluorophenylacetylene as the alkyne provided **5e** with a conversion of 86% in 14 h (entry 5), while 2-methoxyphenylacetylene afforded **5f** with a conversion of 90% in 14 h (entry 6). On the other hand, the use of 3-methylphenylacetylene as the alkyne gave propargylamine **5g** with a conversion of 93% in 8 h (entry 7). In the reaction of benzaldehyde, 1,3-diethynylbenzene, and piperidine in the presence of Au–SILP (0.1 mol% Au) at 60 °C over 8 h, only the monopropargylamine **5h** was formed (83% conversion, entry 8). Morpholine proved to be as reactive as piperidine. The coupling of this amine with benzaldehyde and trimethylsilylacetylene over 8 h furnished **5i** with 97% conversion (entry 9), whereas the analogous reaction using phenylacetylene as the alkyne formed propargylamine **5j** with a conversion of 98% (entry 10). Chiral propargylic amines **5k**,**l** were synthesized with high levels of conversion and excellent diastereoselectivity using (*S*)-2-(methoxymethyl)pyrrolidine as a chiral reagent. Thus, **5k** and **5l** were obtained with conversions of 99% (de 97%, entry 11) and 87% (de 98%, entry 12), respectively.

The catalyst was easily reused up to five times without taking any precautions after taking up the supernatant layer and washing with *n*-pentane. As shown in Table 3, a steady decrease in catalytic activity was observed after every cycle. This result might have arisen from the partial removal of the gold-containing IL layer from the silica surface or the Au(0)NPs from the SiO_2_–IL support.

To check this hypothesis, the supernatant layer was filtered through a membrane filter (pore diameter of 7 µm) after the reaction had been completed, and the mixture was analyzed with ICP-MS. The analysis showed that a very small amount of Au (3.14 ppb) was released in the reaction medium. This result is not consistent with the loss of activity of the catalyst due to gold leaching, but instead supports the partial elimination of the IL from the solid support as being the major responsible of the conversion decrease after every reaction cycle [82].

**Scheme 2:**
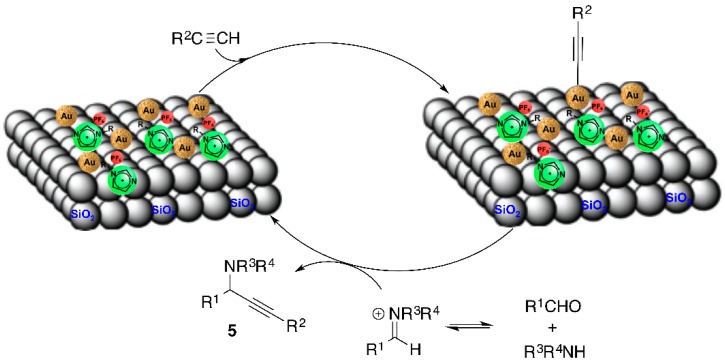
Suggested mechanism for the A^3^ coupling synthesis of propargylamine **5** catalyzed by Au–SILP.

A tentative reaction mechanism for the synthesis of propargylamine **5** through the condensation of an aldehyde, an acetylene, and an amine involving the immobilized AuNPs is shown in Scheme 2. The nanoparticles may activate the alkyne to generate the corresponding supported alkynyl–gold complex, which, upon addition to the iminium ion proceeding from the in situ reaction between the aldehyde and the secondary amine, would provide propargylamine **5** and regenerate the catalyst for a new cycle.

### 2.2. Covalent Bonding Via Ionic Liquid Fragments Attached to the Support

**Scheme 3:**
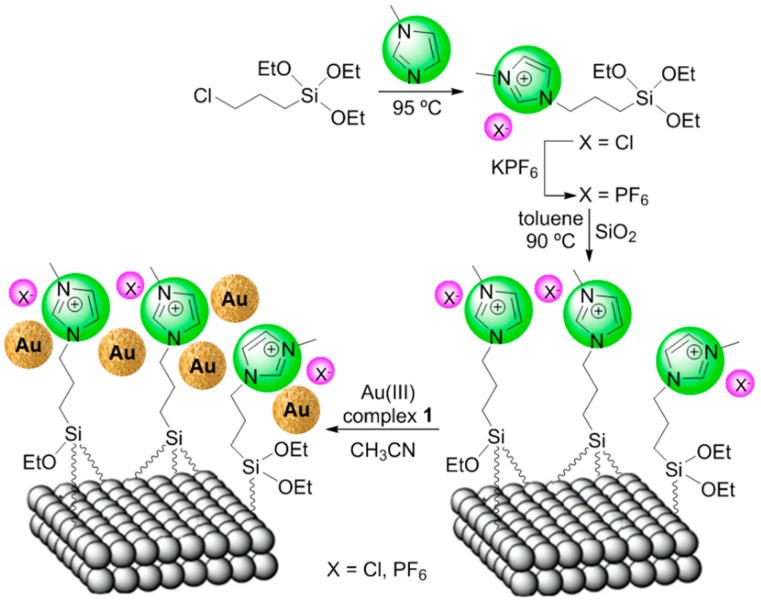
Grafting of the ionic liquid (IL) onto the silica surface and preparation of the immobilized catalyst.

In order to avoid the detachment of the ionic liquid layer from the silica surface in the subsequent reaction cycles, we next investigated the provision of strong attachment of the ionic liquid to the silica surface via covalent bonds. According to the procedure described in the literature [89], the (EtO)_3_Si modified IL, either with chloride or with hexafluorophosphate anions, was immobilized on the silica by the reaction of an alkoxy group bonded to the Si atom of the IL with an Si–OH group of the silica material, forming covalent –Si–O–Si– bonding (Scheme 3). Then, a suspension of the silica with the covalently attached IL (SiO_2_@IL) in a solution of (dppta)AuCl_2_ complex **1** in CH_3_CN was evaporated to dryness and washed with diethyl ether to give a powdery immobilized catalyst. The supported Au(III) complex on SiO_2_@IL(PF_6_) [(dppta)AuCl_2_-SiO_2_@IL(PF_6_)] was characterized by IR and HRMAS NMR spectroscopy.

The IR spectrum revealed the presence of bands at wavelengths of 3442 cm^−1^ and 1132 cm^−1^ assigned to the Si−OH stretch and the Si−O−Si stretch, respectively. The presence of the ionic liquid is clearly indicated by the bands at 3117 cm^−1^ and 3165 cm^−1^, attributed to the C−H ring stretching vibration of the imidazolium, and also by the bands at 1632 cm^−1^ and 846 cm^−1^, assigned to the C=N stretching vibration and to the stretching vibration of PF_6_^−^, respectively (Figure S10). The ^1^H and ^13^C HRMAS NMR spectra of (dppta)AuCl_2_-SiO_2_@IL(PF_6_) measured with DMSO-*d*_6_ as the solvent showed the same features as the Au physisorbed catalyst (Figures S11 and S12). The ^1^H- and ^13^C-NMR spectra could be assigned without difficulty (see experimental section). The very broad ^1^H and ^13^C signals observed for the CH_2_SiO moiety reflect a decrease in mobility, which support the formation of an Si–O bond with the solid support. More importantly, although the IL:**1** ratio (moles) of 85:1 in (dppta)AuCl_2_–SiO_2_@IL(PF_6_) was much bigger than that in the SILP system, the ^31^P HRMAS NMR spectrum could be used to establish the loading of **1** into the IL. The integrals of the signals of PF_6_^-^ and (dppta)AuCl_2_ indicated that complex **1** was quantitatively dispersed into the IL (Figure S15). Due to the high dilution of complex **1** into the IL, 8192 scans were accumulated over a period of 5 h to achieve an acceptable signal-to-noise ratio.

In order to compare the performance of the Au catalyst immobilized on the ionic liquid and covalently grafted to the silica surface (Au–SiO_2_@IL) vs. the physically adsorbed analogue (Au-SILP), we carried out the A^3^ coupling reaction of benzaldehyde, piperidine, and phenylacetylene at 60 °C for 8 h with a catalyst loading of 0.1%. Under these conditions, the corresponding propargylamine was obtained with 98% conversion, which is comparable to the results with the confined catalyst. In order to check the reusability, the catalyst was repeatedly filtered out and subjected to a new reaction batch without any further treatment. The recyclability of a catalyst is highly dependent on the counter anion of the IL. Thus, for the chloride IL, a sharp decrease in catalytic activity was observed in the second reaction cycle. In the third cycle, the chloride catalyst hardly showed any activity, whereas the hexafluorophosphate catalysts showed only a very small decrease in the benzaldehyde conversion (Table 4). The dependence of the physicochemical properties and activity of the imidazolium ILs on the anion is not surprising and has been widely reported in the literature [90,91].

**Figure 4:**
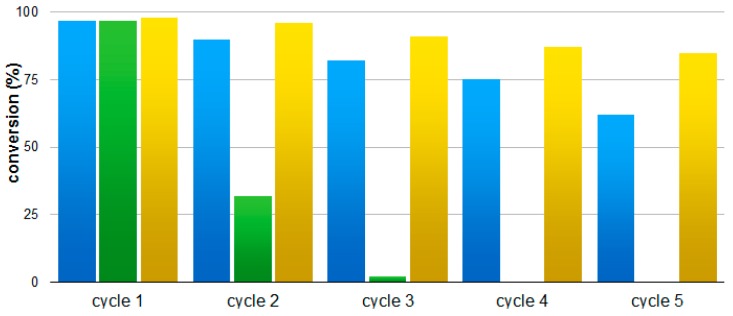
Recyclability as a function of the catalyst. Blue: Au–SILP; green: Au–SiO_2_@IL(Cl); yellow: Au–SiO_2_@IL(PF_6_).

The catalytic activity of the hexafluorophosphate catalyst was high over five reaction cycles, so the recyclability of the covalently grafted supported phase catalyst was clearly superior to the physically adsorbed support (Figure 4).

**Figure 5:**
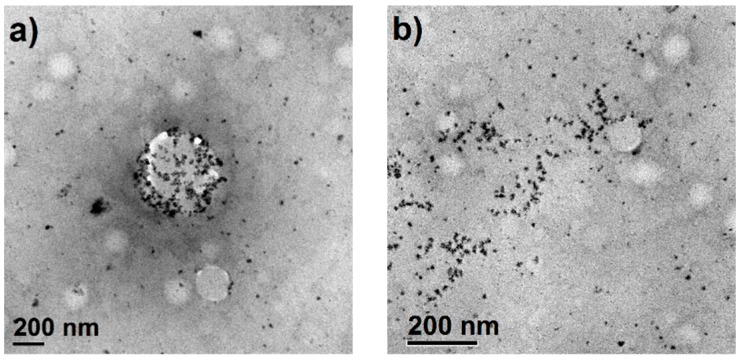
Transmission electron microscopy (TEM) (**a**) and (**b**) (larger surface area) images of AuNPs formed during the A^3^ synthesis of **5a** in the presence of 0.1 mol% of (dppta)AuCl_2_–SiO_2_@IL(PF_6_) under solvent-free conditions.

The Au–SiO_2_@IL(PF_6_) catalyst was characterized using transmission electron microscopy and X-ray photoelectron spectroscopy methods. The TEM image showed the AuNPs immobilized on the IL-coated silica (Figure 5) with a narrow size distribution with a diameter of about 7.8 nm, comparable to the physically adsorbed supported system (Figure 2).

**Figure 6:**
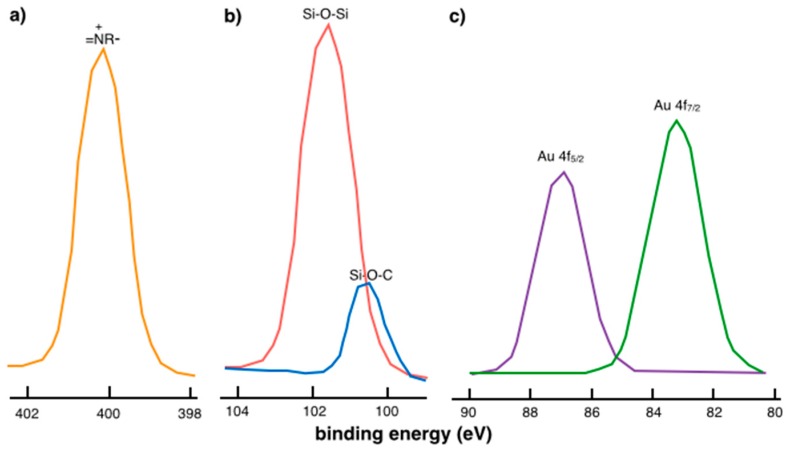
XPS spectra of the core level regions of Au–SiO_2_@IL(PF_6_) for (**a**) N 1s, (**b**) Si 2p, and (**c**) Au 4f.

In the full XPS spectrum of the Au–SiO_2_@IL(PF_6_) catalyst, the expected peaks corresponding to Au 4f, C 1s, Cl 2s, N 1s, O 1s, and Si 2p and 2s were observed (Figure S16). The XPS spectrum of the N 1s core level region for Au–SiO_2_@IL(PF_6_) showed a peak at 400.0 eV, corresponding to the bonding energy of the quaternary nitrogen of IL that was clearly observed (Figure 6a and Figure S17). The high resolution Si 2p XPS spectrum of the Au–SiO_2_@IL(PF_6_) could be split into two sublevels. The first one at 101.6 eV was assigned to the Si–O–C bonds, while the other one at 102.9 eV corresponded to the silicon participant from the siloxane network (Si–O–Si) (Figure 6b and Figure S18). Finally, the XPS spectrum in the Au 4f region showed two intense doublets at 83.6 and 87.3 eV, comprising the only Au species in the material and confirming that, during the catalysis, the ionic Au(III) species were reduced (Figure 6c and Figure S19).

## 3. Conclusions

In summary, we developed an environmentally benign, economically friendly, and sustainable A^3^ coupling reaction of alkynes, aldehydes, and amines by employing a cycloaurated phosphinothioic amide gold(III) complex immobilized in a silica-supported ionic liquid. During the course of the reaction, gold(0) nanoparticles were formed in situ and were subsequently confined to the SiO_2_–IL phase. The AuNPs immobilized in this way maintained good catalytic activity for 3–4 cycles, but then a decrease in conversion was observed. This loss of activity can be attributed to the progressive detachment of the ionic liquid from the silica surface. In order to overcome this limitation, the ILs were grafted onto the silica surface by means of covalent bonds. Due to the more robust anchors that prevented Au–IL leaching from the silica support, the reusability of the resulting Au–SiO_2_@IL catalyst was clearly enhanced, maintaining high activity for up to five reaction cycles. In addition, ^31^P HRMAS NMR spectroscopy was used for the first time to establish the loading of Au into the ionic liquid phase.

## 4. Materials and Methods

Benzaldehyde, piperidine, morpholine, phenylacetylene, and trimethylsilylacetylene were obtained through commercial suppliers and purified by distillation before use. The rest of the reagents were commercially purchased and used without further purification. Amorphous silica from Merck was used as solid support (spherical for flash column chromatography; 40~50 mm diameter; 5~7 nm pore size; 0.80~1.00 mL/g pore volume; 600~700 m^2^/g surface area). Compounds **5a**, [92] **5b** [81], **5c** [81], **5d** [93], **5e** [94], **5f** [95], 5**g** [95], **5h** [81], 5**i** [9], **5j** [94], **5k** [81], and **5l** [81] have been described previously. NMR spectra were obtained on a Bruker Avance III HD 300 (^1^H, 300.13 MHz; ^13^C, 75.47 MHz; ^31^P, 121.49 MHz) and Bruker Avance III HD 500 (^1^H, 500.13 MHz; ^13^C, 125.76 MHz; ^31^P, 202.46 MHz). Chemical shifts are given in ppm using tetramethylsilane (TMS) for ^1^H and ^13^C and 85% H_3_PO_4_ for ^31^P as internal standards. Unless otherwise stated, ^1^H- and ^31^P-NMR spectra were acquired from all crude reaction mixtures, in CDCl_3_ as solvent. Diastereoselectivities were determined by integration of the ^1^H-NMR spectra of the crude reaction mixtures. The following abbreviations are used to indicate the multiplicity of signals: bs, broad signal; h, heptuplet. Regarding HRMAS NMR spectroscopy, the spectra were acquired using a 4 mm ^1^H/^13^C/^31^P HRMAS probehead on a Bruker Avance III HD 500. The MAS rate used was 4600 Hz. A typical ^1^H HRMAS NMR spectrum consisted of 32 transients using 52 K data points over a 11,000 Hz spectral width. For ^13^C HRMAS NMR spectra, 16,000 transients were acquired using 64 K data points over 25,252 Hz spectral widths. ^31^P HRMAS NMR spectra were acquired using 64 K data points over 59,524 Hz spectral widths and an accumulation of 2–8 K. The ^1^H, ^31^P HMQC spectrum was acquired using the “hmqcgpqf” pulse program. Selected spectral parameters were as follows: spectral width, 8711 Hz for ^1^H and 30,748 Hz for ^31^P; 128 increments recorded; cnst2 *^n^J*_PH_ = 8.3 Hz; and 1024 scans per increment in F1. Two-dimensional double quantum-filtered ^1^H, ^1^H COSY45° spectra were recorded using the pulse program “cosy45gpqf” with 128 increments and 64 scans. An edited two-dimensional ^1^H, ^13^C HSQC spectrum was acquired using the “hsqcedetgpsp.3” pulse program including adiabatic pulses for selection with 160 increments and 128 scans.

### 4.1. TEM and XPS Measurements

The size and distribution of AuNPs formed in the reaction were studied by transmission electron microscopy (TEM) using a JEOL-2100 TEM (Peabody, MA, USA) instrument operating at 200 kV fitted with an Orius SC 200 Model 830 (Gatan Inc., Pleasanton, CA, USA) camera. Samples were prepared by placing ca. 15 μL of the solution onto a Formvar- and carbon-coated copper grid. The samples were allowed to dry and introduced in the instrument. For the statistical particle size analysis, 524 nanoparticles were analyzed using the measurement tools of the Digital Micrograph v2.31.734.0 (Gatan Microscopy Suite, Gatan Inc., Pleasaton, CA, USA) software package. Data processing and statistical studies were performed with Excel Office 2010 (Microsoft Corporation., Redmond, WA, USA). X-ray photoelectron spectroscopy (XPS) was carried out on an ESCAPlus Omicron spectrometer (Omicron Nanotechnology, Taunusstein, Germany) using a monochromated Mg X-ray source (1253.6 eV). The binding energy scale was calibrated by setting the C 1s transition to 284.7 eV. Data were analyzed using the Multipak XPS software package (Physical Electronics Inc., Chanhassen, MN, USA).

### 4.2. Scanning Electron Microscopy

Scanning electron microscopy (SEM) was done on a Hitachi S-3500N (Hitachi Ltd., Tokyo, Japan) operated at 15 kV with a tungsten filament. Powder from each sample was placed on a stub and coated with a 10 nm gold layer using a BAL-TEC SCD 005 Sputter Coater (BalTec Corporation, Canonsburg, PA, USA) to obtain the images using a secondary electron detector.

### 4.3. Representative Procedure for Catalyst Preparation

Method A: Silica powder (1 g) was added to a stirred solution of (dppta)AuCl_2_ complex **1** (12 mg) in [bmim]PF_6_ (0.1 g) and CH_3_CN (10 mL). After being stirred for 90 min at room temperature, CH_3_CN was evaporated to dryness to give a light yellow dry powder which was rinsed with diethyl ether and dried in vacuo to afford the Au-supported catalyst (~1% wt based on weight gain and ^31^P HRMAS NMR). Spectroscopic data for the pre-catalyst (dppta)AuCl_2_–SiO_2_–[bmim]PF_6_ was as follows (molar ratio Au:IL 1:19, the (dppta)AuCl_2_ was assigned with the aid of 2D HRMAS NMR experiments): ^1^H HRMAS NMR (500 MHz, DMSO-*d*_6_): δ = 0.85 (bs, 3H, CH_2_C*H*_3_), 1.14 (bs, 6H, CH(C*H*_3_)_2_), 1.19 (bs, 6H, CH(C*H*_3_)_2_), 1.73 (bs, 2H, CH_2_), 3.65 (bs, 6H, C*H*(CH_3_)_2_), 3.80 (bs, 3H, NCH_3_), 4.1 (bs, 2H, CH_2_N), 7.39 (bs, 1H, NCHC) 7.43 (bs, 1H, NCHC), 7.43 (bs, 1H, ArH), 7.46 (bs, 1H, ArH), 7.62 (bs, 1H, ArH), 7.70 (bs, 2H, ArH), 7.74 (bs, 1H, ArH), 8.13 (bs, 2H, ArH), 8.17 (bs, 1H, ArH), 8.43 (bs, 1H, NCHN) ppm; ^13^C HRMAS NMR (125.8 MHz, DMSO-*d*_6_): δ = 13.6 (CH_3_), 19.2 (CH_2_), 23.0 (CH(*C*H_3_)_2_), 31.7 (CH_2_), 36.1 (NCH_3_), 49.0 (NCH_2_), 50.7 (*C*H(CH_3_)_2_) 122.7 (CHN), 123.9 (NCH), 128.4 (CHAr), 129.9 (CHAr), 132.8 (CHAr), 129.9 (2 × CHAr), 133.2 (CHAr), 133.3 (2 × CHAr), 134.9 (CHAr), 135.1 (CHAr), 136.8 (NCN) ppm; ^31^P HRMAS NMR (202.5 MHz, DMSO-*d*_6_): δ = −144.1 (h, ^1^*J*_PF_ 706.2 Hz), 68.9 ppm.

Method B: 1-(Triethoxysilylpropyl)-3-methyl-imidazolium chloride and hexafluorophosphate were prepared as reported in the literature [96]. Subsequently, the ILs were grafted over the silica surface giving rise to SiO_2_@IL(Cl) and SiO_2_@IL(PF_6_), respectively. The procedure used was as follows: 1 g of the silica was placed into a round-bottom flask and firstly heated under reduced pressure with a heat-gun; then, a solution containing 1.5 g of ionic liquid in 5 mL of dry toluene was added and the mixture was stirred at 90 °C for 16 h. After cooling, the solid was filtered. Then, to a stirred solution of (dppta)AuCl_2_ complex **1** (12 mg) in CH_3_CN (10 mL), 1.1 g of either SiO_2_@IL(Cl) or SiO_2_@IL(PF_6_), was added. After being stirred for 90 min at room temperature, CH_3_CN was evaporated to dryness to give a light yellow dry powder which was rinsed with diethyl ether and dried in vacuo to afford the Au-supported catalysts Au–SiO_2_@IL(Cl) and Au–SiO_2_@IL(PF_6_), respectively (~1% wt based on weight gain and ^31^P HRMAS NMR). The spectroscopic data for the pre-catalyst (dppta)AuCl_2_–SiO_2_@IL(PF_6_) was as follows (molar ratio Au:IL 1:85, not possible to assign the signals of the (dppta)AuCl_2_ complex due to the very low relative proportion): ^1^H HRMAS NMR (500 MHz, DMSO-*d*_6_): δ = 0.51 (bs, 2H, SiCH_2_), 1.78 (bs, 2H, CH_2_), 3.88 (bs, 3H, NCH_3_), 4.16 (bs, 2H, CH_2_N), 7.69 (bs, 1H, NCHC) 7.84 (bs, 1H, NCHC), 9.36 (bs, 1H, NCHN) ppm; ^13^C HRMAS NMR (125.8 MHz, DMSO-*d*_6_): δ = 8.8 (SiCH_2_), 23.9 (CH_2_), 36.1 (NCH_3_), 51.2 (NCH_2_), 122.5 (CHN), 123.8 (NCH), 136.9 (NCN) ppm; ^31^P HRMAS NMR (202.5 MHz, DMSO-*d*_6_): δ = −144.1 (h, ^1^*J*_PF_ 706.2 Hz), 68.8 ppm.

### 4.4. General Procedure for the Gold-Catalyzed, Three-Component Coupling

A mixture of aldehyde (1.97 mmol), amine (1.97 mmol), acetylene (2.95 mmol), and the corresponding supported Au catalyst (1% wt, 60 mg, 0.002 mmol) was heated at 60 °C for 8 h, after which time the solution was cooled and the catalyst was removed by filtration. The filtrate was evaporated under reduced pressure to afford propargylamine **5**. Yields were determined by integration of the ^1^H-NMR spectra of the crude reaction mixtures. After separation and washing with *n*-pentane, the catalyst was reused intact for the next reaction without any further pre-treatment. Representative examples of the isolated products are as follows.

*1-(1-Phenyl-3-(trimethylsilyl)prop-2-ynyl)piperidine* (**5a**). Prepared according to the general procedure. Purified by column chromatography on silica gel (3% EtOAc in hexanes). Colorless oil, 81% yield (0.432 g, 1.59 mmol). ^1^H-NMR (300.13 MHz, CDCl_3_): δ 0.29 (s, 9H), 1.43−1.49 (m, 2H), 1.56−1.67 (m, 4H), 2.48−2.52 (m, 4H), 4.63 (s, 1H), 7.29−7.40 (m, 2H, ArH), 7.61 (d, 2H, *J =* 7.5 Hz, ArH).

*1-(3-(2-Methoxyphenyl)-1-phenylprop-2-yn-1-yl)piperidine* (**5f**). Prepared according to the general procedure. Purified by column chromatography on silica gel (5% EtOAc in hexanes). Colorless oil, 82% yield (0.492 g, 1.61 mmol). ^1^H-NMR (300.13 MHz, CDCl_3_): δ 1.48−1.53 (m, 2H), 1.62−1.68 (m, 4H), 2.63−2.66 (m, 4H), 3.94 (s, 3H), 4.91 (s, 1H), 6.92-6.99 (m, 2H, ArH), 7.31−7.43 (m, 4H, ArH), 7.52−7.56 (m, 1H, ArH), 7.75 (d, 2H, *J =* 7.5 Hz, ArH).

*1-(3-(3-Ethynylphenyl)-1-phenylprop-2-ynyl)piperidine* (**5h**). Prepared according to the general procedure. Purified by column chromatography on silica gel (5% EtOAc in hexanes). Colorless oil, 80% yield (0.478 g, 1.58 mmol). ^1^H-NMR (300.13 MHz, CDCl_3_): δ 1.46−1.49 (m, 2H), 1.60−1.65 (m, 4H), 2.56−2.60 (m, 4H), 3.12 (s, 1H), 4.82 (s, 1H), 7.29−7.42 (m, 4H, ArH), 7.46−7.53 (m, 2H, ArH), 7.64−7.68 (m, 3H, ArH).

*(S)-2-(Methoxymethyl)-1-(1-phenyl-3-(trimethylsilyl)prop-2-yn-2-yl)pyrrolidine* (**5k**). Purified by column chromatography on silica gel (5% EtOAc in hexanes). Colorless oil, 81% yield (0.486 g, 1.59 mmol). ^1^H-NMR (300.13 MHz, CDCl_3_) δ: 0.26 (s, 9H), 1.59−1.75 (m, 3H), 1.89−1.98 (m, 1H), 2.49−2.53 (m, 1H), 2.63−2.71 (m, 1H), 3.20−3.28 (m, 1H), 3.39−3.54 (m, 2H), 3.43 (s, 3H), 5.11 (s, 1H), 7.25−7.38 (m, 3H, ArH), 7.58−7.61 (m, 2H, ArH).

## Figures and Tables

**Table 1:**
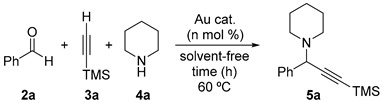
Optimization of the A^3^ coupling reaction of benzaldehyde, trimethylsilylacetylene, and piperidine.

Entry	Catalyst	*n* (mol%)	Time (h)	Conv. (%) ^a^
1	(dppta)AuCl_2_–SILP	1	6	99
2	(dppta)AuCl_2_–SILP	0.5	6	98
3	(dppta)AuCl_2_–SILP	0.1	8	97
4	(dppta)AuCl_2_	1	6	97
5	(dppta)AuCl_2_	0.1	8	99

^a^ Determined by ^1^H-NMR analysis of the reaction crude.

**Table 2:**
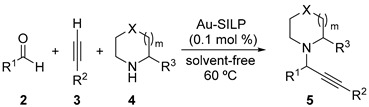
A^3^ coupling reaction catalyzed by Au–SILP.

Entry	R^1^	R^2^	R^3^	X	m	Time (h)	5	Conv. (%) ^a^
1	Ph	TMS	H	CH_2_	1	8	a	97
2	4-ClC_6_H_4_	TMS	H	CH_2_	1	8	b	94
3	4-OMeC_6_H_4_	TMS	H	CH_2_	1	14	c	83
4	Ph	Ph	H	CH_2_	1	8	d	94
5	Ph	4-FC_6_H_4_	H	CH_2_	1	14	e	86
6	Ph	2-OMeC_6_H_4_	H	CH_2_	1	14	f	90
7	Ph	3-MeC_6_H_4_	H	CH_2_	1	8	g	93
8	Ph	3(C≡C)C_6_H_4_	H	CH_2_	1	8	h	83
9	Ph	TMS	H	O	1	8	i	97
10	Ph	Ph	H	O	1	8	j	98
11	Ph	TMS	CH_3_	CH_2_	0	8	k	98(97) ^b^
12	Ph	Ph	CH_3_	CH_2_	0	8	l	87(98) ^b^

^a^ Conversion (conv.) determined by ^1^H-NMR analysis of the crude reaction. ^b^ Values in parentheses indicate diastereomeric excesses, de. SILP: supported ionic liquid phase catalysis.

**Table 3:** Reusability of the Au–SILP catalyst.

Entry	Cycle	Conv. (%) ^a^
1	1	97
2	2	90
3	3	81
4	4	75
5	5	62

^a^ Determined by ^1^H-NMR analysis of the reaction crude.

**Table 4:**
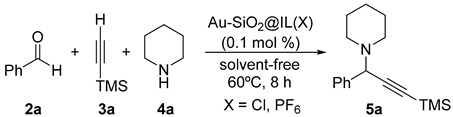
A^3^ coupling reaction catalyzed by Au–SiO_2_@IL and the recycling of the catalyst.

Entry	Catalyst	Conversion (%) ^a^				
		Cycle 1	Cycle 2	Cycle 3	Cycle 4	Cycle 5
1	Au–SiO_2_@IL(Cl)	97	32	0	-	-
2	Au–SiO_2_@IL(PF_6_)	98	96	91	86	85

^a^ Determined through ^1^H-NMR analysis of the reaction crude.

## Supplementary Materials

The Supplementary Materials are available online. Figure S1: IR spectrum of the pre-catalyst (dppta)AuCl_2_–SiO_2_–[bmim]PF_6_. Figure S2: ^1^H HRMAS NMR spectrum of the pre-catalyst (dppta)AuCl_2_-SiO_2_-[bmim]PF_6_. Figure S3: ^13^C HRMAS NMR spectrum of the precatalyst (dppta)AuCl_2_-SiO_2_–[bmim]PF_6_. Figure S4: ^1^H, ^1^H COSY HRMAS NMR spectrum of the pre-catalyst (dppta)AuCl_2_–SiO_2_–[bmim]PF_6_. Figure S5: ^1^H, ^13^C HSQC-Edited HRMAS NMR spectrum of the pre-catalyst (dppta)AuCl_2_–SiO_2_–[bmim]PF_6_. Figure S6: Full XPS spectrum of the catalyst Au–SiO_2_–[bmim]PF_6_. Figure S7: Core level region XPS spectra of N 1s of the catalyst Au–SiO_2_–[bmim]PF_6_. Figure S8: Core level region XPS spectra of Si 2p of the catalyst Au–SiO_2_–[bmim]PF_6_, Figure S9: Core level region XPS spectra of Au 4f of the catalyst Au–SiO_2_–[bmim]PF_6_, Figure S10: IR spectrum of the pre-catalyst (dppta)AuCl_2_–SiO_2_@IL(PF_6_), Figure S11: ^1^H HRMAS NMR spectrum of the pre-catalyst (dppta)AuCl_2_–SiO_2_@IL(PF_6_), Figure S12: ^13^C HRMAS NMR spectrum of the pre-catalyst (dppta)AuCl_2_–SiO_2_@IL(PF_6_), Figure S13: ^1^H, ^1^H gCOSY HRMAS NMR spectrum of the pre-catalyst (dppta)AuCl_2_–SiO_2_@IL(PF_6_), Figure S14: ^1^H, ^13^C HSQC-edited HRMAS NMR spectrum of the pre-catalyst (dppta)AuCl_2_–SiO_2_@IL(PF_6_), Figure S15: ^31^P HRMAS NMR spectrum of the pre-catalyst (dppta)AuCl_2_–SiO_2_@IL(PF_6_), Figure S16: Full XPS spectrum of the catalyst Au–SiO_2_@IL(PF_6_), Figure S17: Core level region XPS spectra of N 1s of the catalyst Au–SiO_2_@IL(PF_6_), Figure S18: Core level region XPS spectra of Si 2p of the catalyst Au–SiO_2_@IL(PF_6_), Figure S19: Core level region XPS spectra of Au 4f of the catalyst Au–SiO_2_@IL(PF_6_).
