# Supplementary material for: Immobilized Gold Nanoparticles Prepared from Gold(III)-Containing Ionic Liquids on Silica: Application to the Sustainable Synthesis of Propargylamines
Source: Molecules. 2018 Nov 14;23(11):2975. doi: 10.3390/molecules23112975 (PMC6278337; doi:10.3390/molecules23112975)
Supplement: Supplementary file 1 [file molecules-23-02975-s001.pdf]

Supporting Information

Immobilized gold nanoparticles prepared from gold(III)-containing ionic liquids on silica: application to the sustainable synthesis of propargylamines

Raquel Soengas,* Yolanda Navarro, María José Iglesias and Fernando López Ortiz*

Área de Química Orgánica, Research Centre CIAIMBITAL, Universidad de Almería, Ctra. Sacramento s/n, 04120 Almería, Spain

Contents

IR spectrum of the precatalyst (dppta)AuCl$_2$-SiO$_2$-[bmim]PF$_6$.......................................S2
Figure S1. FT-IR spectrum.
HRMAS NMR spectra of the precatalyst (dppta)AuCl$_2$-SiO$_2$-[bmim]PF$_6$....................S3
Figure S2. ^{1}H HRMAS NMR spectrum.
Figure S3. ^{13}C HRMAS NMR spectrum.
Figure S4. ^{1}H,^{1}H COSY HRMAS NMR spectrum.
Figure S5. ^{1}H,^{13}C HSQC-edited HRMAS spectrum.
XPS of the catalyst Au-SiO$_2$-[bmim]PF$_6$...S5
Figure S6. Full XPS spectrum.
Figure S7. Core level region XPS spectra of N 1s.
Figure S8. Core level region XPS spectra of Si 2p.
Figure S9. Core level region XPS spectra of Au 4f.
IR of the precatalyst (dppta)AuCl$_2$-SiO$_2$@IL(PF$_6$)...S7
Figure S10. FT-IR spectrum.
HRMAS NMR spectra of the precatalyst (dppta)AuCl$_2$-SiO$_2$@IL(PF$_6$)..........................S8
Figure S11. ^{1}H HRMAS NMR spectrum.
Figure S12. ^{13}C HRMAS NMR spectrum.
Figure S13. ^{1}H,^{1}H COSY HRMAS NMR spectrum.
Figure S14. Edited ^{1}H,^{13}C HSQC HRMAS NMR spectrum.
Figure S15. ^{31}P NMR HRMAS NMR spectrum.
XPS spectra of the catalyst Au-SiO$_2$@IL(PF$_6$)...S11
Figure S16. Full XPS spectrum.
Figure S17. Core level region XPS spectra of N 1s.
Figure S18. Core level region XPS spectra of Si 2p.
Figure S19. Core level region XPS spectra of Au 4f.

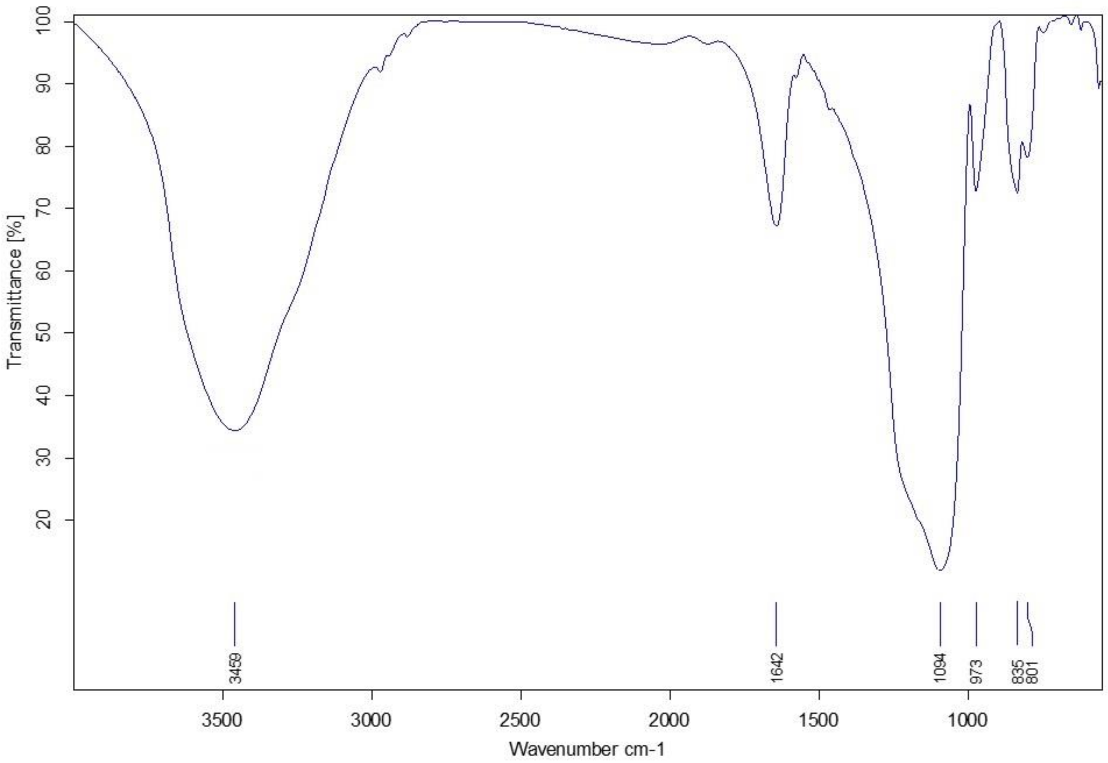

Figure S1. FT-IR spectrum of the precatalyst (dppta)$AuCl_2$-SiO_2-[bmim]PF_6 measured in KBr.

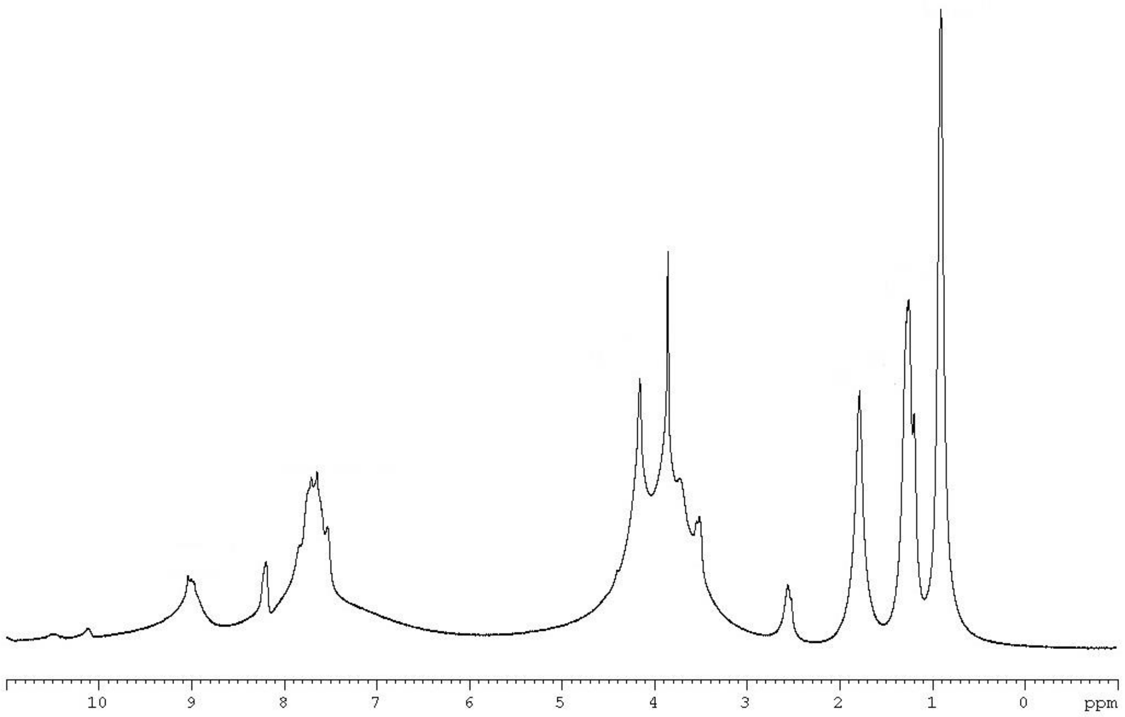

Figure S2. ^{1}H HRMAS NMR spectrum (500.13 MHz) of the precatalyst (dppta)$AuCl_2$-SiO_2-[bmim]PF_6 measured in DMSO-d_6.

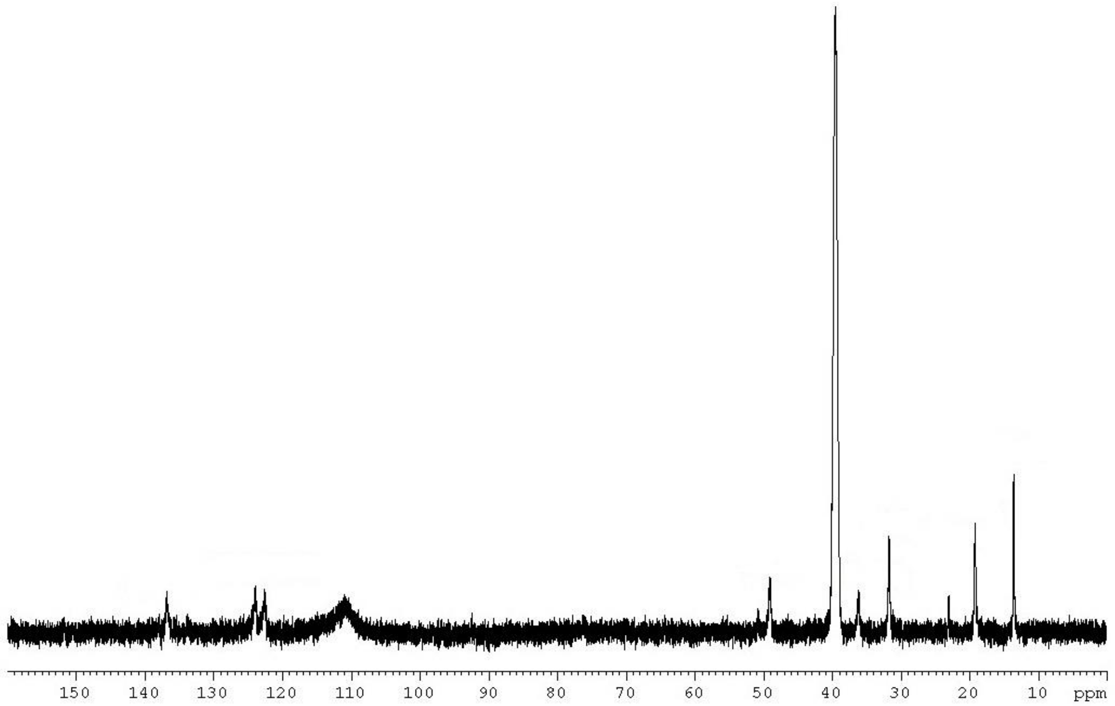

Figure S3. ^{13}C HRMAS NMR spectrum (125.76 MHz) of the precatalyst (dppta)$AuCl_2$-SiO_2-[bmim]PF_6 measured in DMSO-d_6.

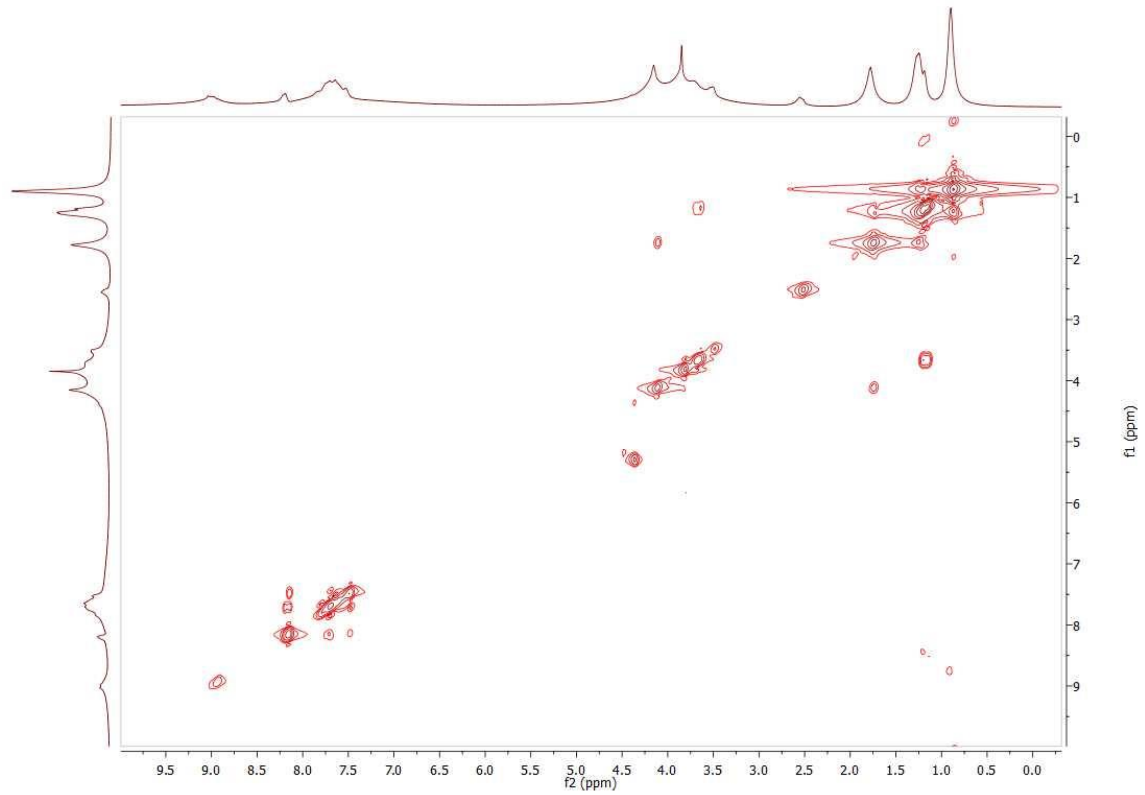

Figure S4. $^{1}H,^{1}H$ COSY HRMAS NMR spectrum (500.13 MHz) of the precatalyst (dppta)$AuCl_2$-SiO_2-[bmim]PF_6 measured in DMSO-d_6.

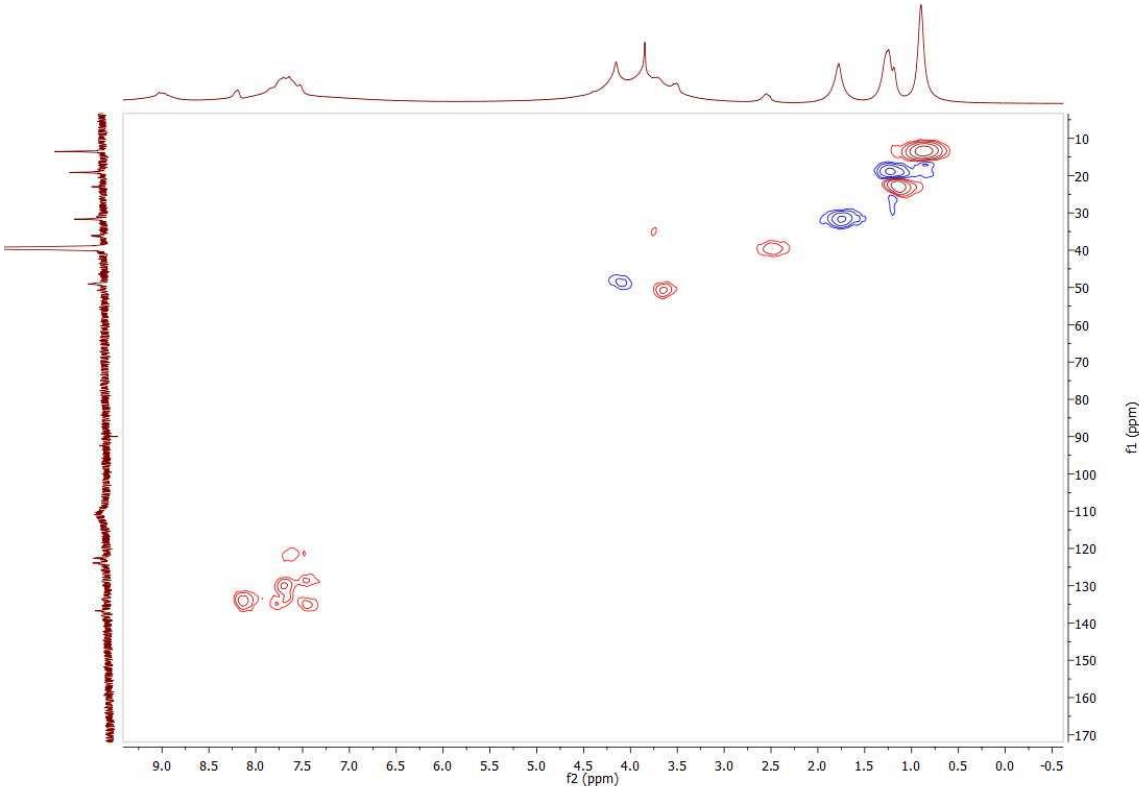

Figure S5. Edited $^{1}H,^{13}C$ HSQC HRMAS NMR spectrum (500.13 MHz) of the precatalyst (dppta)$AuCl_2$-SiO_2-[bmim]PF_6 measured in DMSO-d_6.

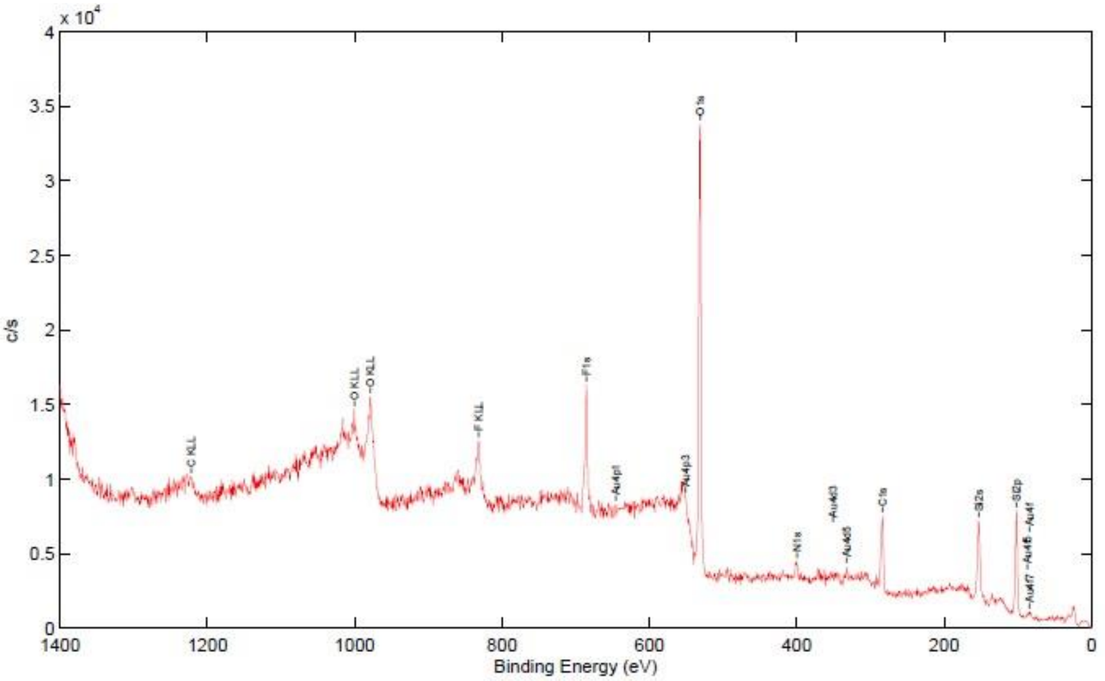

Figure S6. Full XPS spectrum of the catalyst Au-SiO_2-[bmim]PF_6.

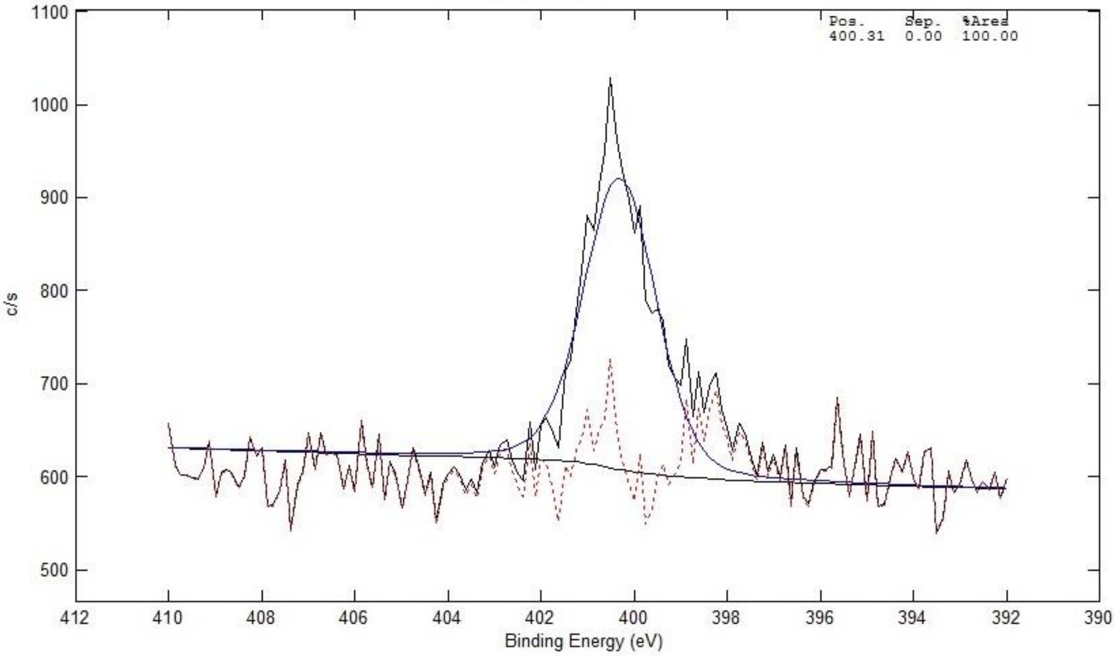

Figure S7. Core level region XPS spectra of N 1s of the catalyst Au-SiO_2-[bmim]PF_6.

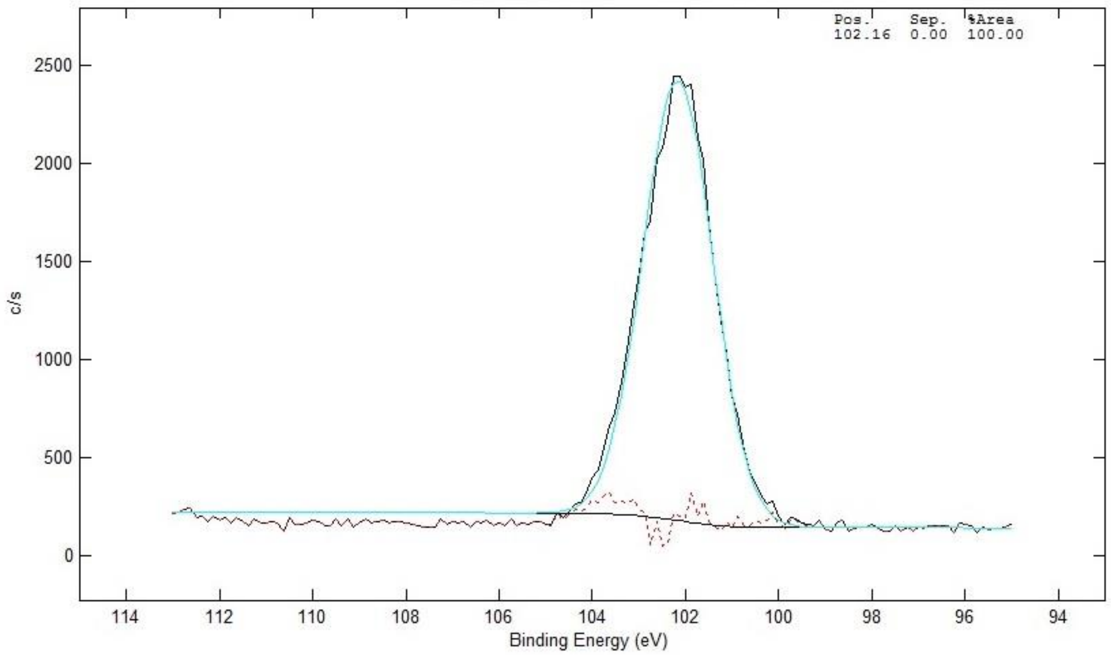

Figure S8. Core level region XPS spectra of Si 2p of the catalyst Au-SiO_2-[bmim]PF_6.

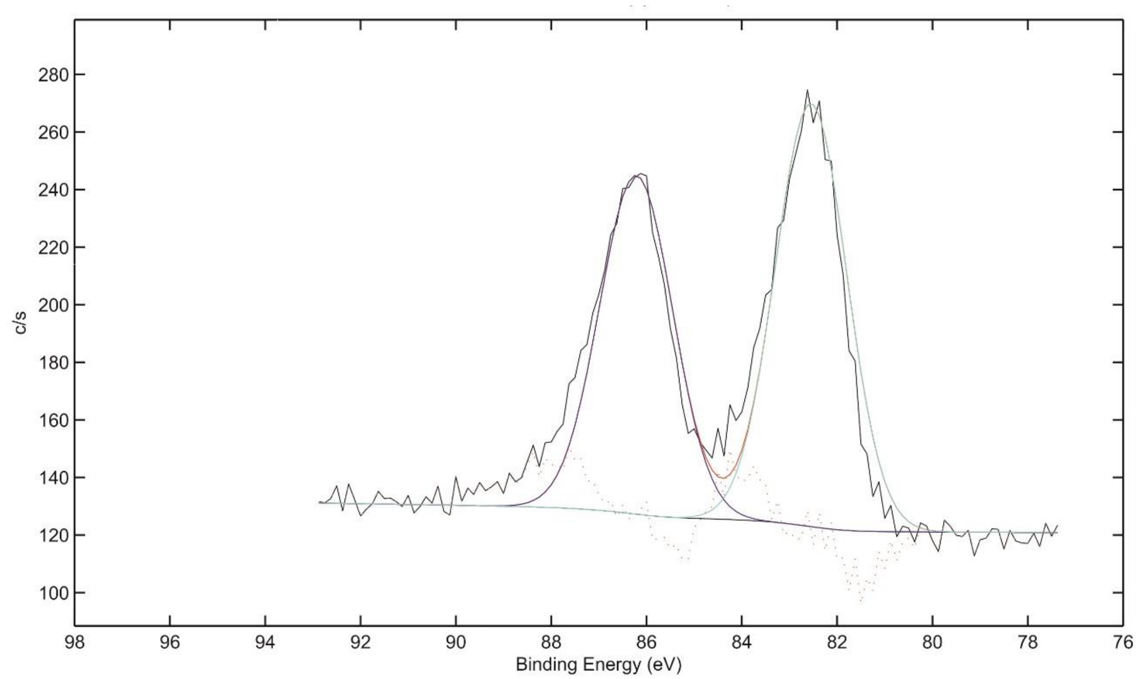

Figure S9. Core level region XPS spectra of Au 4f of the catalyst Au-SiO_2-[bmim]PF_6.

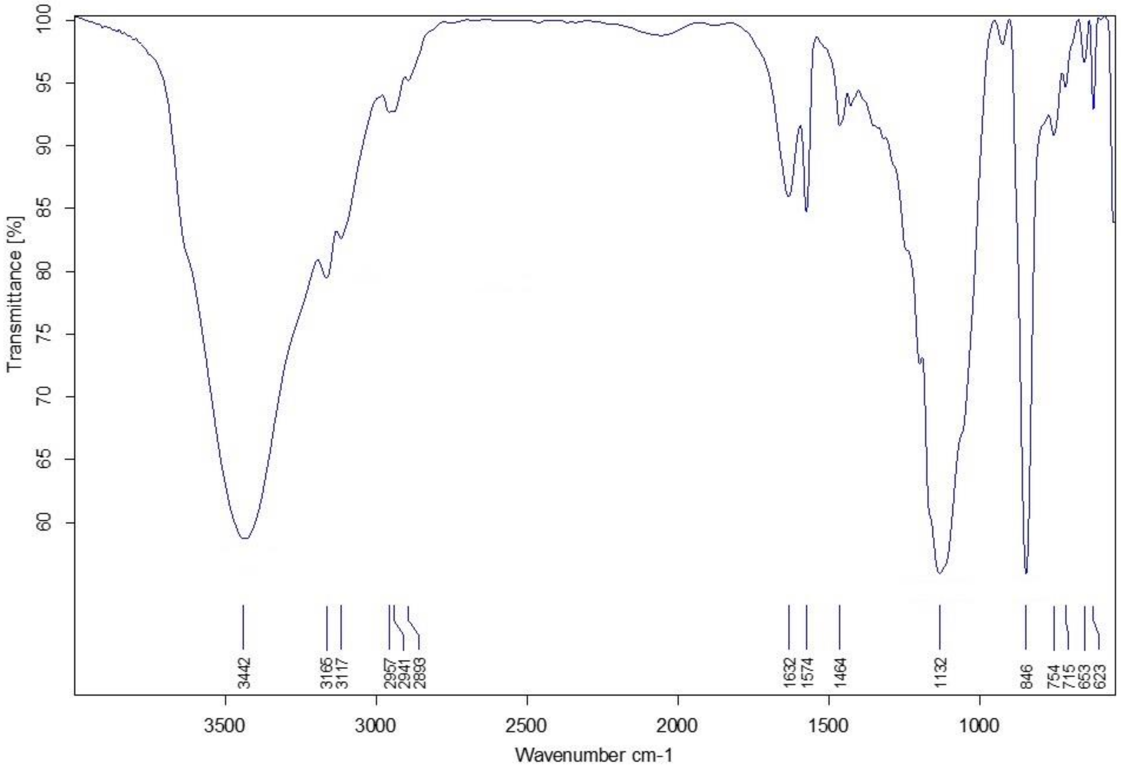

Figure S10. FT-IR of the precatalyst (dppta)$AuCl_2$-SiO_2@IL(PF_6) measured in KBr.

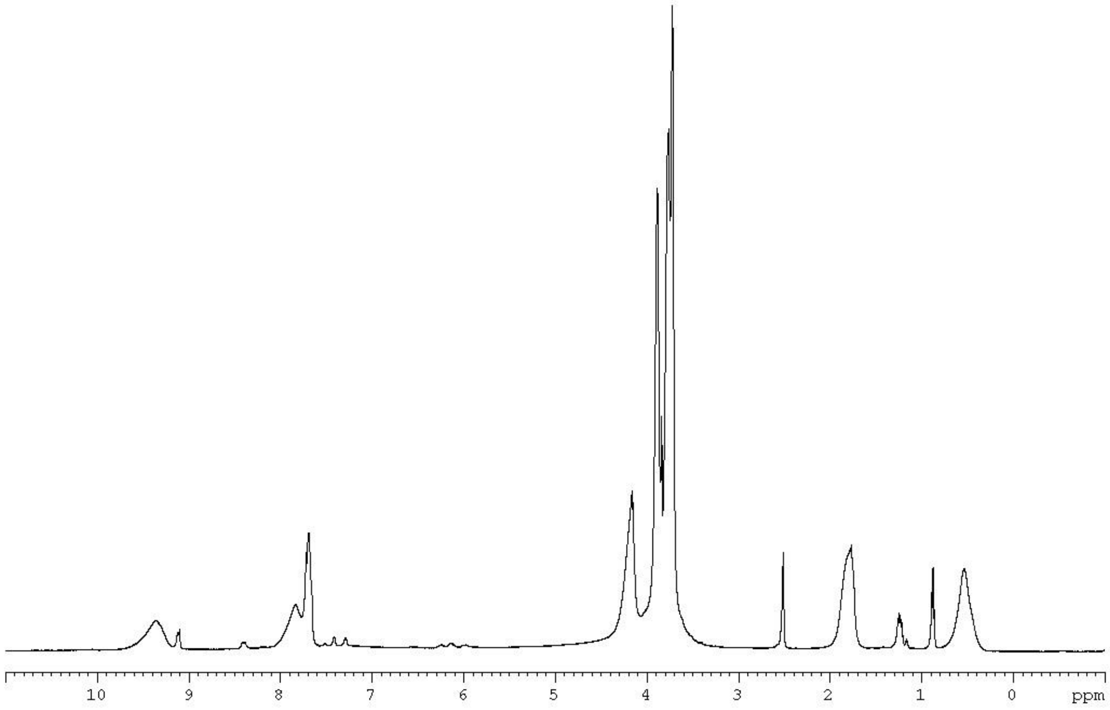

Figure S11. ^{1}H HRMAS NMR spectrum (500.13 MHz) of the precatalyst (dppta)$AuCl_2$-SiO_2@IL(PF_6) measured in DMSO-d_6.

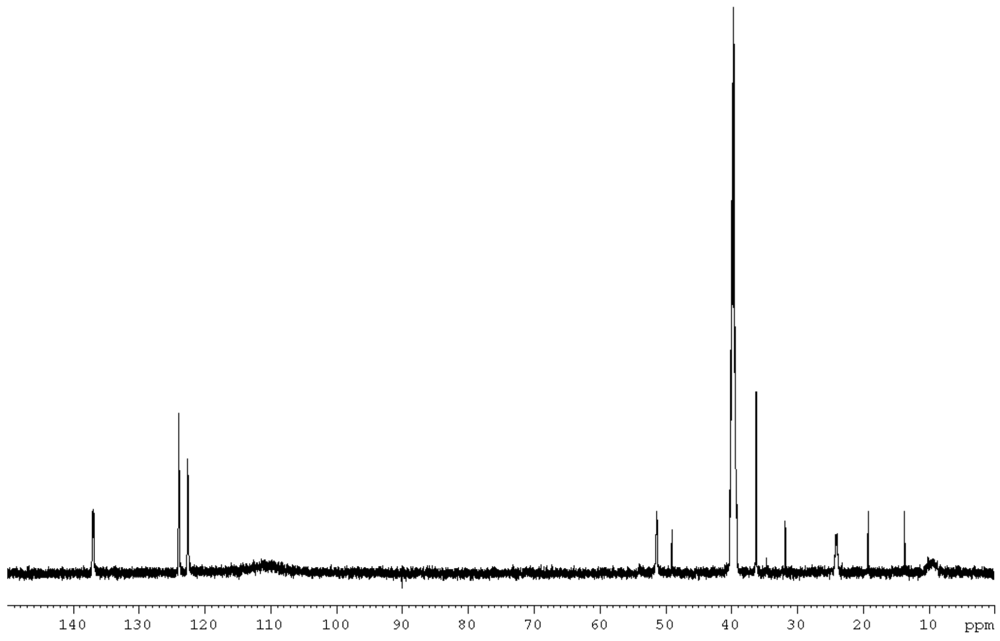

Figure S12. ^{13}C HRMAS NMR spectrum (125.76 MHz) of the precatalyst (dppta)$AuCl_2$-SiO_2@IL(PF_6) measured in DMSO-d_6.

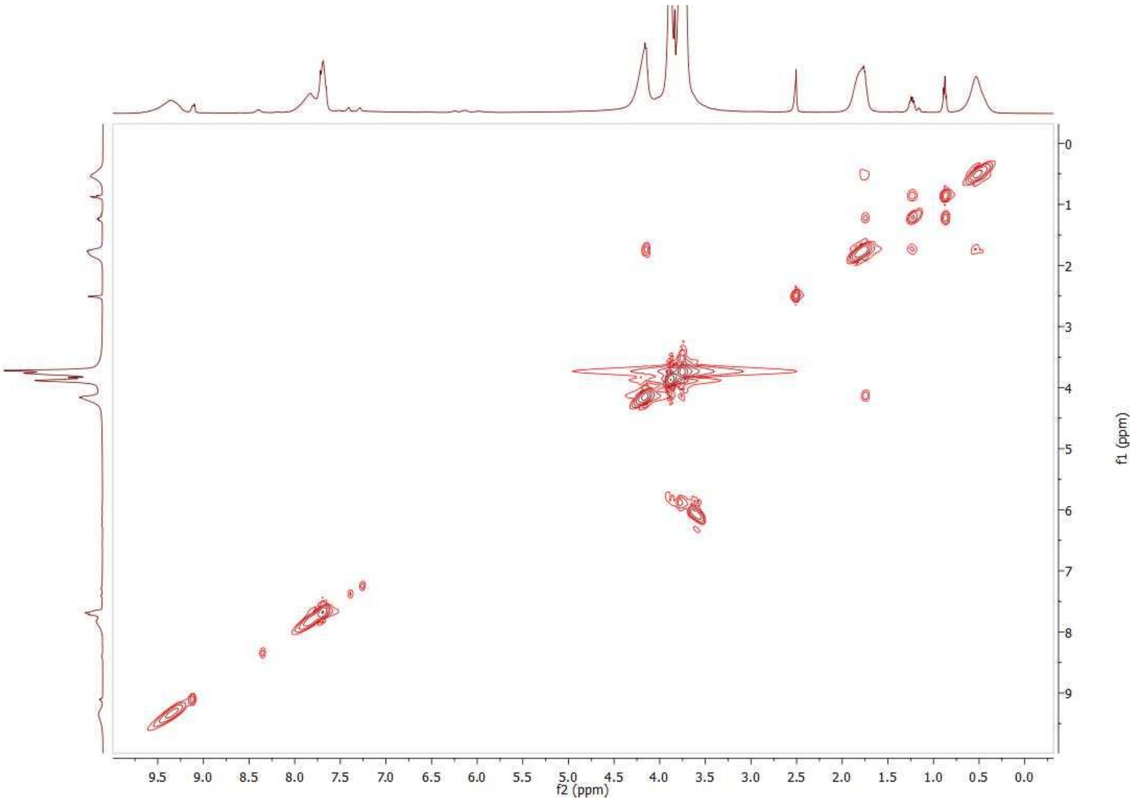

Figure S13. $^{1}H,^{1}H$ COSY HRMAS NMR spectrum (500.13 MHz) of the precatalyst (dppta)$AuCl_2$-SiO_2@IL(PF_6) measured in DMSO-d_6.

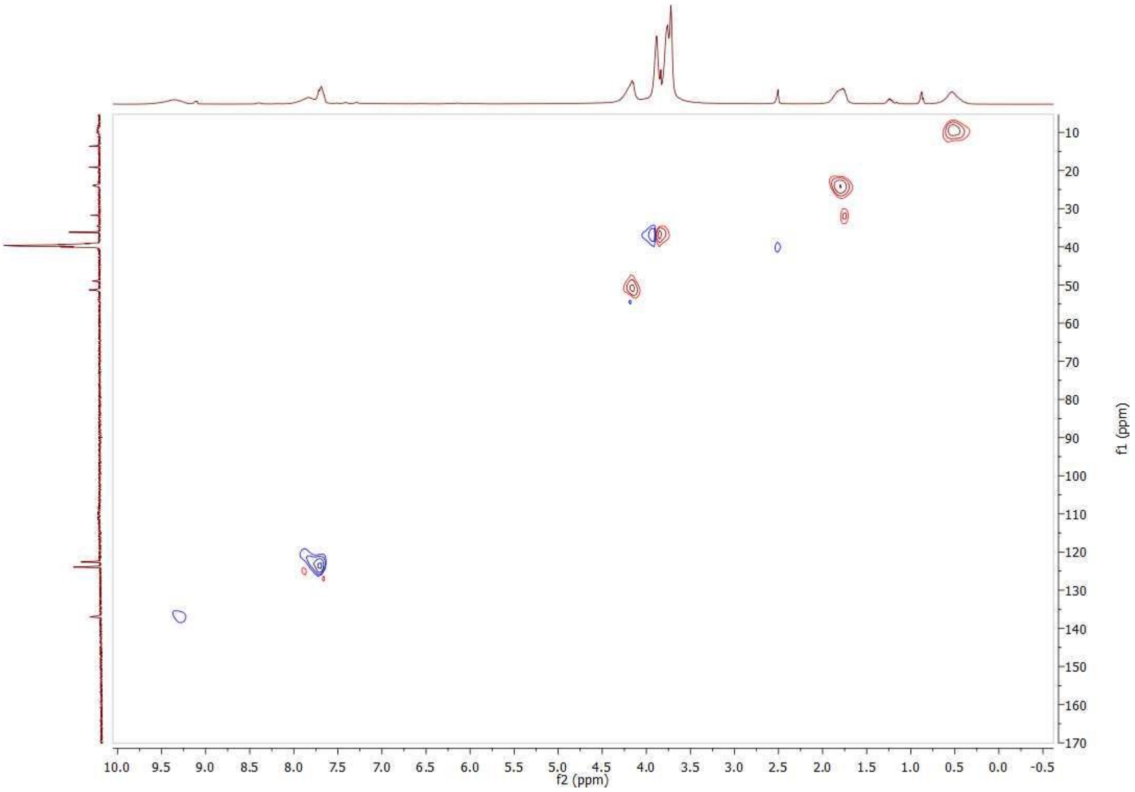

Figure S14. Edited $^{1}H,^{13}C$ HSQC HRMAS NMR spectrum (500.13 MHz) of the precatalyst (dppta)$AuCl_2$-SiO_2@IL(PF_6) measured in DMSO-d_6.

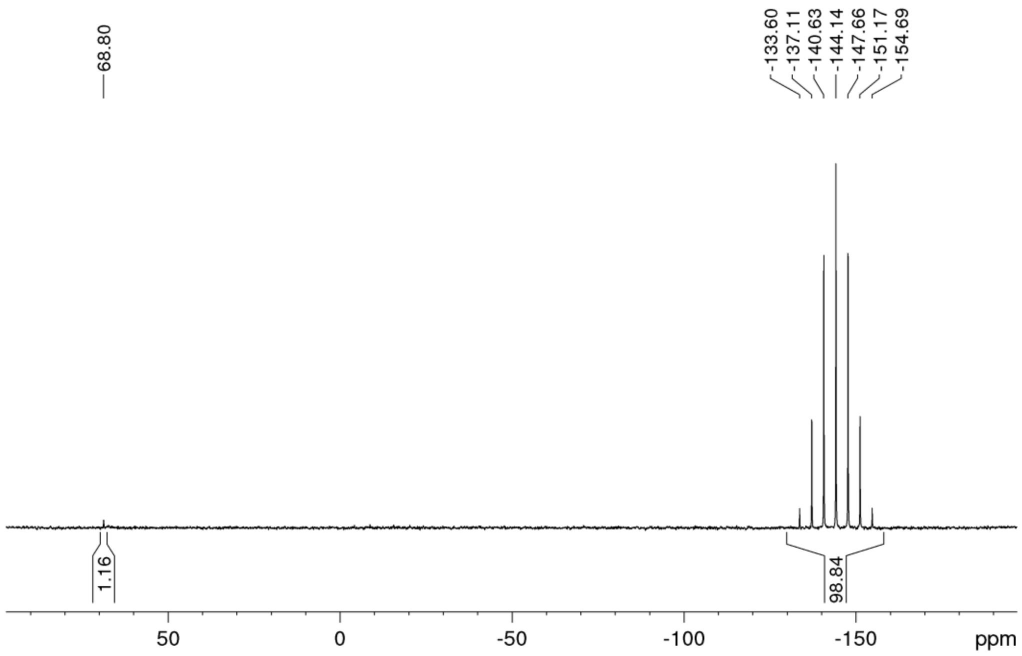

Figure S15. ${}^{31}P$ HRMAS NMR spectrum (202.46 MHz) of the precatalyst (dppta)$AuCl_2$-SiO_2@IL(PF_6) measured in DMSO-d_6.

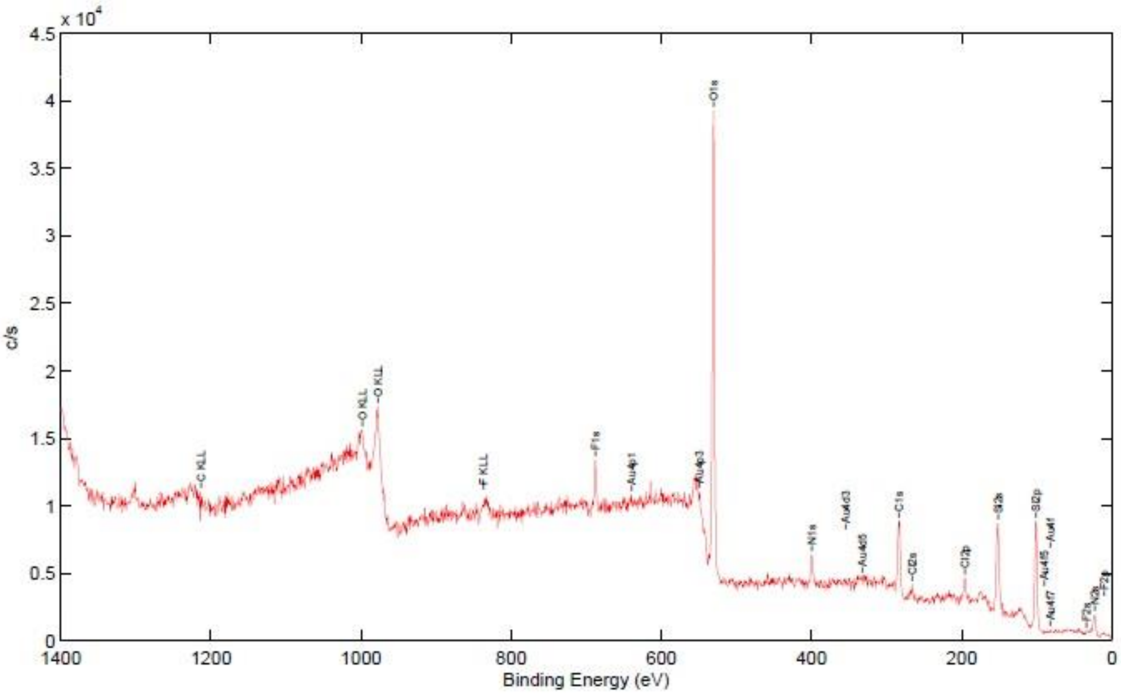

Figure S16. Full XPS spectrum of the catalyst Au-SiO_2@IL(PF_6).

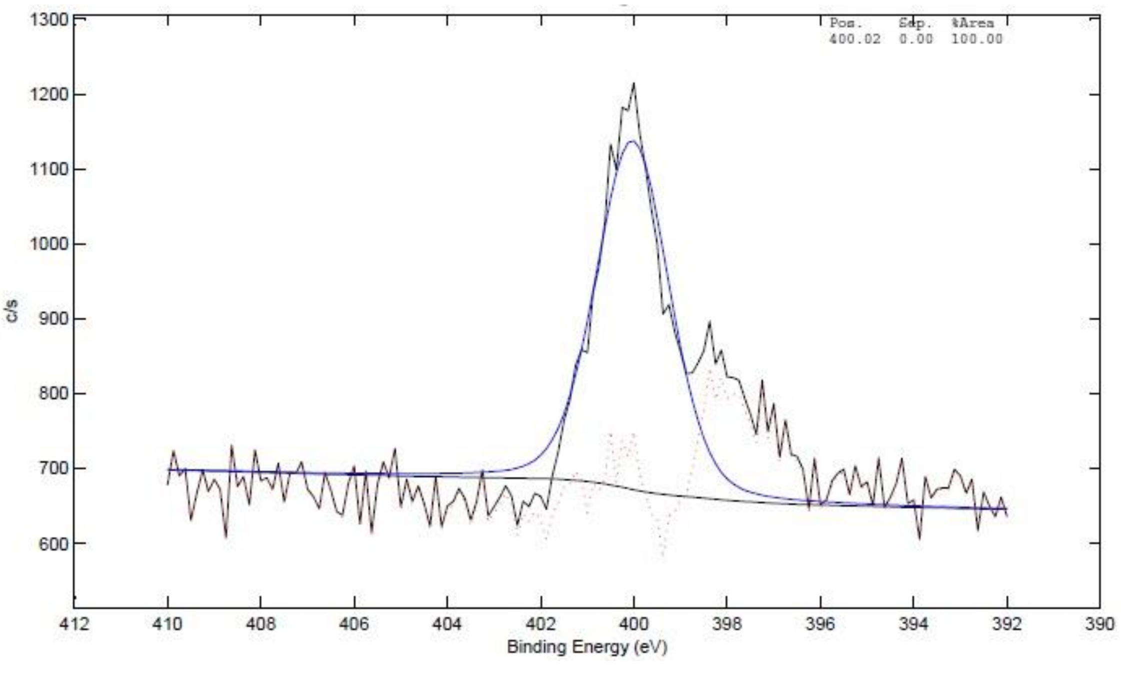

Figure S17. Core level region XPS spectra of N 1s of the catalyst Au-SiO_2@IL(PF_6).

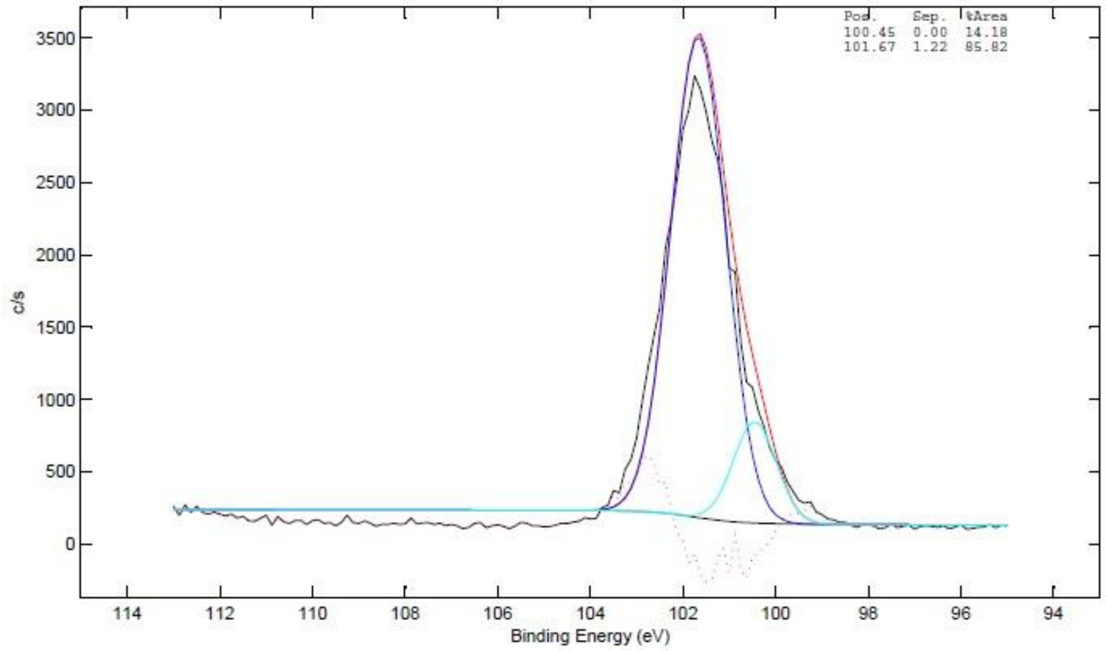

Figure S18. Core level region XPS spectra of Si 2p of the catalyst Au-SiO_2@IL(PF_6).

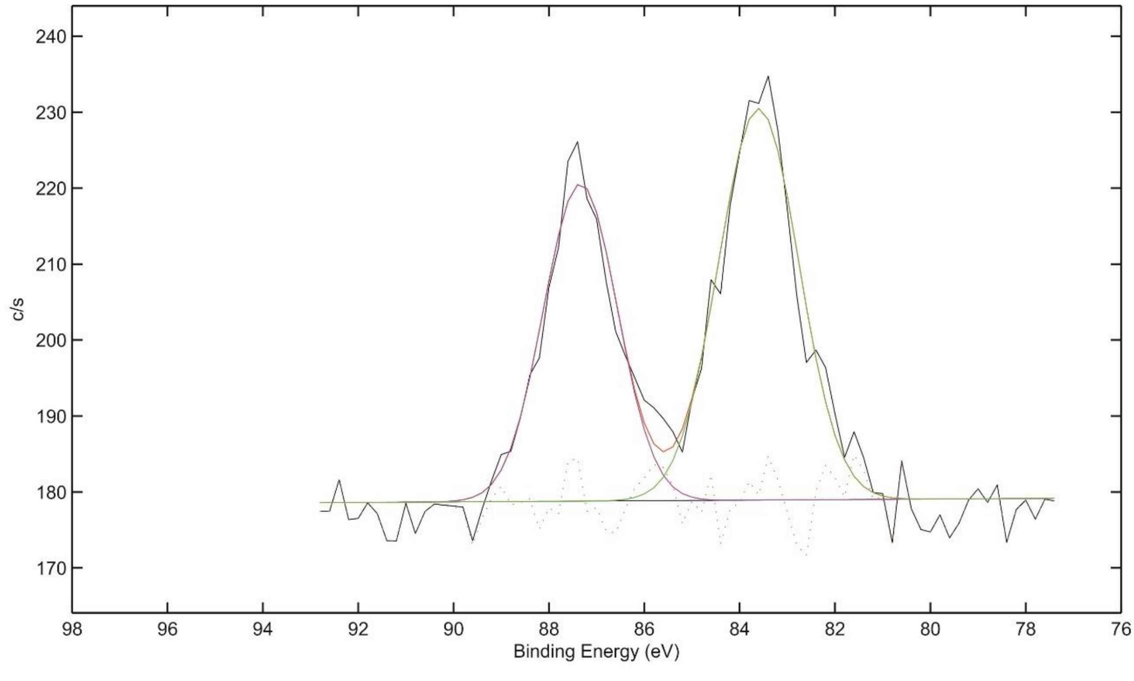

Figure S19. Core level region XPS spectra of Au 4f of the catalyst Au-SiO_2@IL(PF_6).
